# Recent advances in conventional and unconventional vesicular secretion pathways in the tumor microenvironment

**DOI:** 10.1186/s12929-022-00837-8

**Published:** 2022-08-05

**Authors:** I.-Ying Kuo, Chih-Hsiung Hsieh, Wan-Ting Kuo, Chih-Peng Chang, Yi-Ching Wang

**Affiliations:** 1 Department of Pharmacology, College of Medicine, National Cheng Kung University, No.1, University Road, Tainan, 701 Taiwan; 2 Department of Biotechnology, College of Life Science, Kaohsiung Medical University, Kaohsiung, Taiwan; 3 Department of Microbiology and Immunology, College of Medicine, National Cheng Kung University, No.1, University Road, Tainan, 701 Taiwan; 4 Institute of Basic Medical Sciences, College of Medicine, National Cheng Kung University, Tainan, Taiwan

**Keywords:** Rab GTPase, Exocytosis, Extracellular vesicle, Secretory autophagy, Vesicle trafficking, Tumor microenvironment

## Abstract

All cells in the changing tumor microenvironment (TME) need a class of checkpoints to regulate the balance among exocytosis, endocytosis, recycling and degradation. The vesicular trafficking and secretion pathways regulated by the small Rab GTPases and their effectors convey cell growth and migration signals and function as meditators of intercellular communication and molecular transfer. Recent advances suggest that Rab proteins govern conventional and unconventional vesicular secretion pathways by trafficking widely diverse cargoes and substrates in remodeling TME. The mechanisms underlying the regulation of conventional and unconventional vesicular secretion pathways, their action modes and impacts on the cancer and stromal cells have been the focus of much attention for the past two decades. In this review, we discuss the current understanding of vesicular secretion pathways in TME. We begin with an overview of the structure, regulation, substrate recognition and subcellular localization of vesicular secretion pathways. We then systematically discuss how the three fundamental vesicular secretion processes respond to extracellular cues in TME. These processes are the conventional protein secretion via the endoplasmic reticulum-Golgi apparatus route and two types of unconventional protein secretion via extracellular vesicles and secretory autophagy. The latest advances and future directions in vesicular secretion-involved interplays between tumor cells, stromal cell and host immunity are also described.

## Background

Complex and dynamic communication is established between tumor cells and stromal cells in the tumor microenvironment (TME) which is characterized by morphological and functional changes in cancer cells and surrounding stromal cells. These alterations include uncontrolled cancer cell division, proliferation, invasiveness and metastatic ability, as well as the dysregulation of fibroblasts, endothelial cells and infiltrated immune cells [1]. In aggressive TME, malignant cells evade the immune response and establish a very complex balance associated with different immune subtypes [2]. Based on results of recent and ongoing studies, conventional and unconventional vesicular secretion pathways have attracted enormous interest in studying the secretory pathways in the regulation of tumorigenesis and TME.

**Fig. 1:**
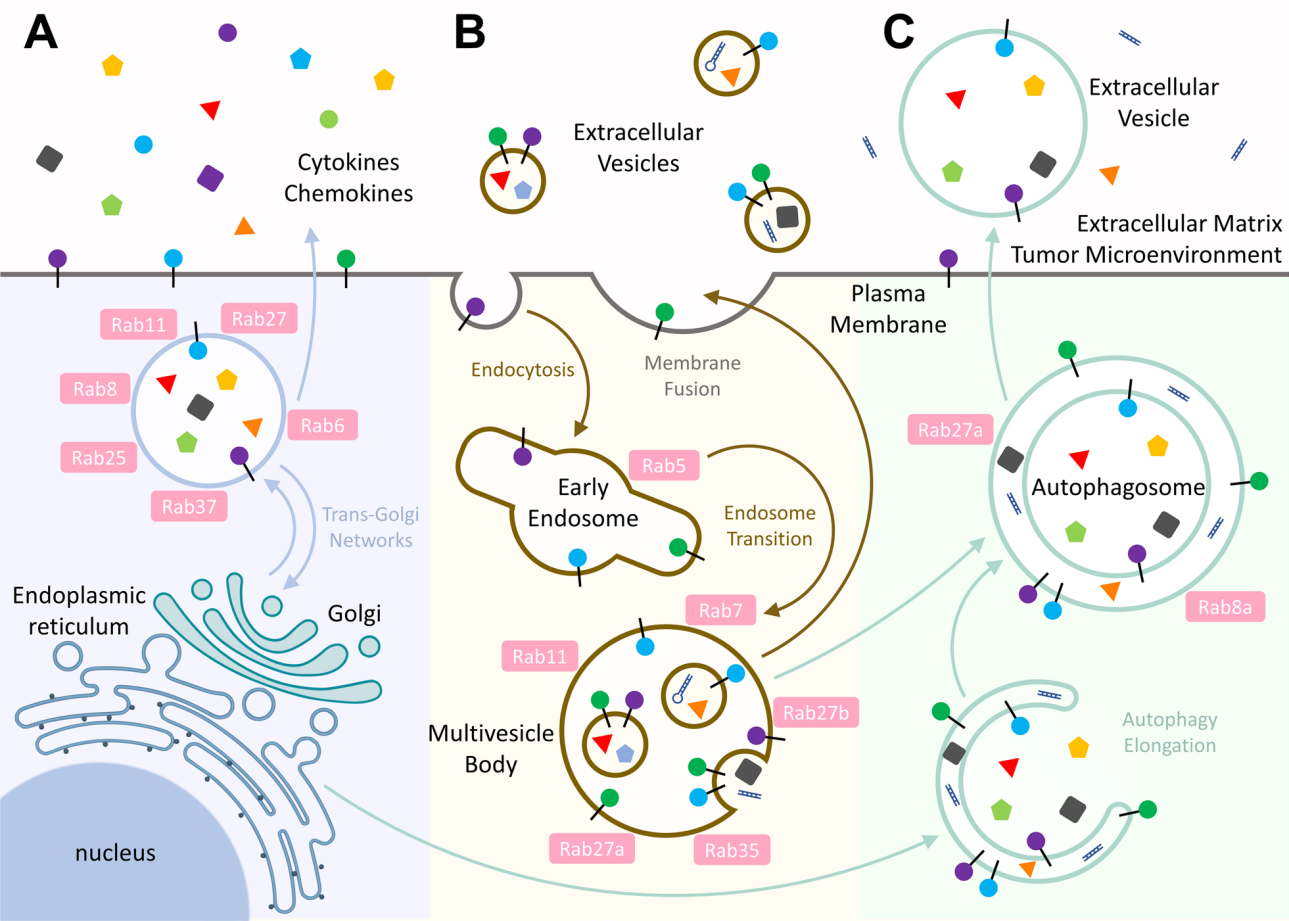
Three fundamental vesicular secretion processes for intercellular communication in TME. **A** Rab GTPases are the key coordinator of membrane trafficking to transport cytokines, chemokines and plasma membrane proteins via the conventional endoplasmic reticulum-Golgi apparatus route. **B** EV, an unconventional protein secretion approach, functions as the critical mediator of intercellular communication, allowing cells to exchange a variety of bioactive molecules. **C** Autophagic molecular machinery has recently been recognized as a novel unconventional protein secretion that plays a part in the molecular symphony being played in TME

Intracellular membrane trafficking is defined as a network of pathways that require transport and exchange of specific cargoes to connect many membrane-bound organelles communicating within the cells as well as between the cells and their environments. In conventional protein secretion (CPS), most secreted proteins require a signal peptide which mediates the co-translational process in endoplasmic reticulum (ER), and then are transported in vesicles to Golgi apparatus (Golgi) followed by constitutive or regulated secretion out of the cell [3]. Rab proteins belong to the largest branch of the Ras superfamily of GTPases. They are membrane attached proteins localized to the cytoplasmic face of organelles and vesicles involved in endocytic and secretory pathways. Rab GTPases are the key regulators of membrane trafficking, although each pathway is controlled by specific regulators. Rab GTPase can coordinate different kinds of responses by recruiting and activating a diverse set of effectors within a distinctive stage of vesicle/membrane traffic, including vesicle budding, delivery, tethering and fusion [2, 4, 5]. Rab proteins also regulate immune responses by controlling transport of immune receptors, secretion of cytokine and chemokine, and upregulating phagocytic capacity for immune surveillance [6–9]. The association of Rab-mediated conventional vesicle traffic in promoting aggressive TME is the focus of the discussion in “Mechanisms of conventional intracellular vesicle trafficking associated with communication between tumor and stromal cells” section (Fig. 1A).

In addition, there are unconventional pathways of protein secretion (UCPS), which bypass the classical ER-Golgi pathway. For example, some proteins are secreted by extracellular vesicles or autophagosomes. Extracellular vesicles (EVs) are a family of lipid bound vesicles secreted by cells into the extracellular environment. These vesicles were initially considered as cellular waste disposal bags. Nevertheless, EVs are now recognized to serve as critical mediators of intercellular communication that allow cells to exchange a variety of bioactive molecules, including proteins, RNAs, DNAs, lipids and metabolites [10]. Hence, the roles of EVs in dynamic interactions between cancer cells and their microenvironment have attracted extensive attention [11]. Indeed, EVs can be generated through (I) direct budding from the plasma membrane (PM) or (II) inward budding of the early endosome membrane to form multivesicular bodies (MVBs), which are subsequently released by exocytosis pathway. During vesicle production, endosomal sorting complex required for transport (ESCRT) system is typically responsible for membrane budding and cargo packing, and several Rab GTPases further mediate the intracellular trafficking of these packages for secretion [12]. Interestingly, bioactive contents of EVs in blood and urine have emerged as novel diagnostic biomarkers for early cancer detection [13]. Furthermore, the natural properties of EVs make them a promising candidate for use as cancer vaccines. In the “Mechanisms of extracellular vesicles associated with communication between tumor and stromal cells” section, we summarize the current knowledge focusing on the biogenesis and biological function of EVs in TME, and therapeutic potentials of EVs (Fig. 1B).

Autophagy is a conserved and catabolic process in which cellular components such as misfolded proteins or damaged organelles are recruited into double-membrane vesicles called autophagosomes. Autophagosomes containing cargoes can fuse with lysosomes to form autolysosomes where cargoes are degraded for cellular recycling and homeostasis in eukaryotes [14]. Interestingly, in addition to the regulation of protein recycling, autophagy machinery has been recently found to participate in the secretion of proteins via type III UCPS pathway. Type III UCPS is also termed as secretory autophagy. This process requires core ATG proteins to facilitate autophagosome formation and cargo engulfment. Cargoes-carrying autophagosomes in UCPS can fuse with endosomes, MVBs or secretory lysosomes and are directly transported to the PM for protein release. Specific cargo receptors and vesicle trafficking proteins are involved in this secretory pathway [15]. Currently, many intracellular proteins have been identified to be released via secretory autophagy to tissues where they regulate crucial physiological as well as pathological conditions. In the “Mechanisms of autophagy-mediated communication between tumor and stromal cells” section, we discuss the molecular mechanisms of secretory autophagy and its regulatory role in TME (Fig. 1C).

## Mechanisms of conventional intracellular vesicle trafficking associated with communication between tumor and stromal cells

In the past two decades, many important reports have linked some Rab proteins to the mechanistic aspects of cancer growth and metastasis. Alteration in the oncogenic Rabs or tumor suppressive Rabs results in cancer promoting properties such as cell proliferation, migration and invasion. Some of them act directly by mediating the vesicular transport of oncogenic factors and others via modulating cancer signaling [4, 16]. The association of Rab-mediated vesicle trafficking with cancer cell proliferation and migration and TME is the focus of the discussion in this section.

### Regulation of cargo trafficking by exocytotic Rab GTPases in cancer cells

**Fig. 2:**
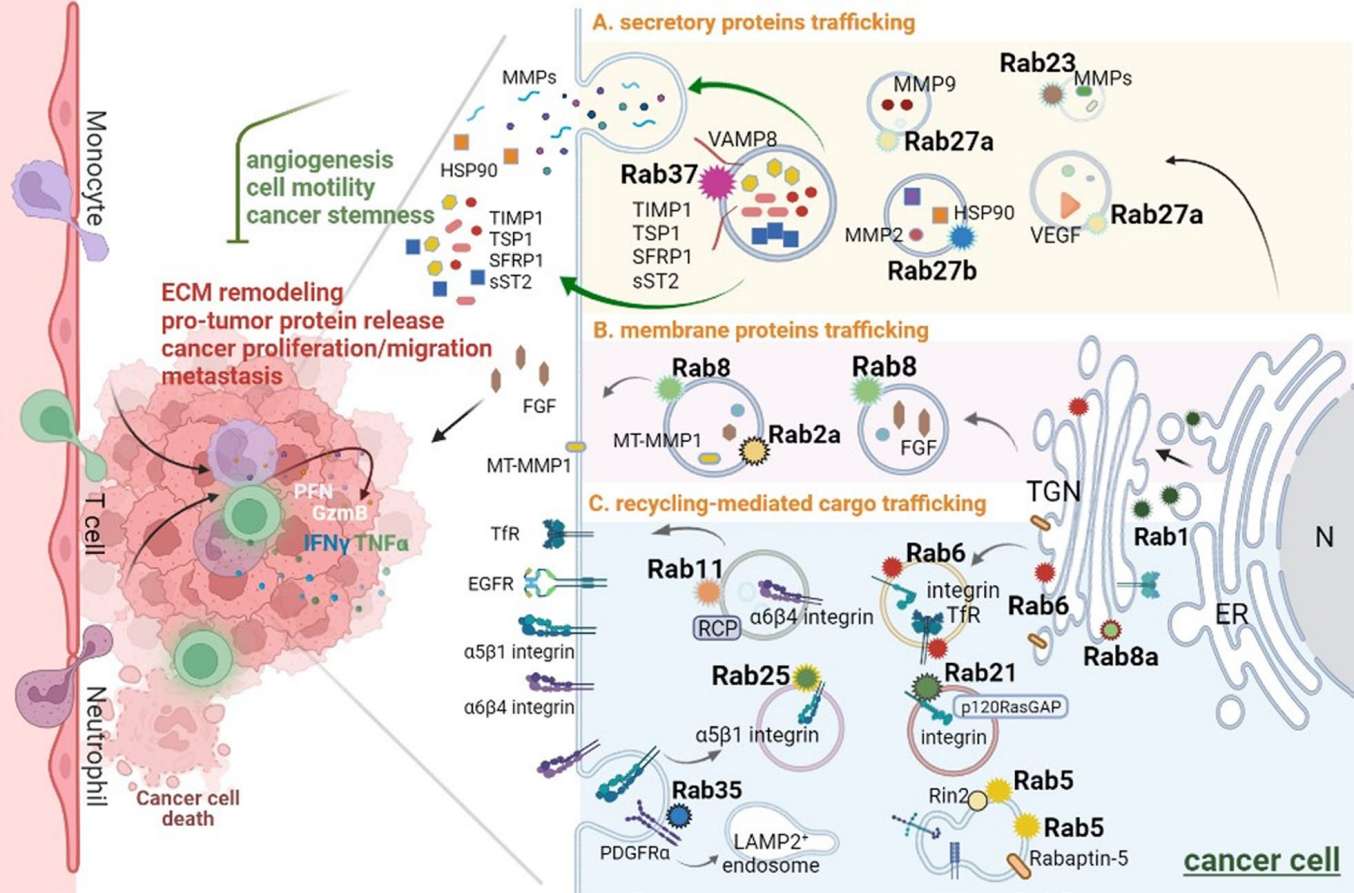
The conventional Rab-specific vesicle trafficking in cancer cells of TME. A wide variety of Rabs are responsible for specific trafficking routes including in endosome, exosome, recycling endosome and lysosome in TME. The specific Rab and its effector(s) regulate conventional secretory vesicle transport of different cargoes in cancer including **A** secretory proteins, **B** membrane proteins and **C** recycling proteins. The Rab-mediated transport of secretory proteins or integrins to the PM results in ECM remodeling, cancer cell proliferation, migration and invasion. Some Rabs act as tumor suppressor-like proteins, such as Rab37. Rab37 mediates secretory proteins transport and exocytosis with the help of the vesicle-SNARE VAMP8. The secreted proteins include TIMP-1, TSP1, SFRP1 and sST2, which inhibit angiogenesis, cell motility and cancer stemness. Please see detailed descriptions about other Rabs in the main text

This review begins by highlighting some of the best-characterized Rab small GTPases and their specific functions, cargoes and interacting regulator/effector proteins in conventional protein secretion (CPS) in cancer cells. Rab proteins are maintained in the GTP-bound active conformation by their regulators guanine exchange factors (GEFs), while GTPase activating proteins (GAPs) stimulate intrinsic GTPase activity of Rabs resulting in an inactive Rab state [17, 18]. In addition, Rab proteins interact with various effector proteins to regulate their respective exocytosis or recycling-mediated secretion pathways. Different effectors act during vesicle formation, movement, tethering and fusion [4, 19–21]. Here we describe some features of Rabs emphasizing on their mechanisms of targeting to specific extracellular cargoes or membrane cargoes (Fig. 2).

#### Exocytic trafficking of secretory proteins in cancer cells

Secretory pathways include transportation of constitutive exocytic vesicles and regulated secretory granules [5, 21, 22]. For example, upon upregulating heat shock protein 90α (HSP90α) trafficking, Rab27b-regulated vesicles are required for matrix metalloproteinase 2 (MMP2) activation to facilitate cancer cell motility [23, 24]. In the glioblastoma, Rab27b mediates the secretion of Epiregulin, which is a membrane protein of the epidermal growth factor (EGF) family, to promote cell proliferation after irradiation treatment in paracrine effects [25]. Rab27a contributes to the invasive and metastatic phenotype in the breast cancer cells by promoting the secretion of insulin-like growth factor-II, which modulates the activity of vascular endothelial growth factor (VEGF), cathepsin D, cyclin D1, urokinase-type plasminogen activator and MMP-9 [26] (Fig. 2A). Additional role of Rab27a/b in EV biogenesis is described in “Mechanisms of extracellular vesicles associated with communication between tumor and stromal cells” section.

Our group has identified several Rab37-regulated secretory proteins modulating extracellular matrix (ECM) and TME. Rab37 mediates the secretion of tissue inhibitor of metalloproteinase 1 (TIMP1), which inhibits the activity of MMP9, to suppress migration, invasion and metastasis in lung cancer. TIMP1 localizes in Rab37-specific vesicles that are transported to extracellular component for exocytosis. Lung cancer patients with low level of Rab37 in tumor tissue show reduced secreted TIMP1 level in intratumor tissue and correlate with distant organ metastasis and poor prognosis [27]. Moreover, thrombospondin 1 (TSP1) is also identified as a cargo protein of Rab37-mediated exocytosis. Rab37 regulates TSP1 secretion in a GTP-dependent manner to inhibit angiogenesis of stromal endothelial cells in TME. Secreted TSP1 from cancer cells with Rab37 exocytic function inhibits the p-focal adhesion kinase (p-FAK)/p-paxillin/p-ERK migration signaling in both cancer epithelial cells and their surrounding endothelial cells. Low Rab37, low TSP1 expression alone with high angiogenesis marker CD31 in lung cancer patients is associated with poor disease outcome [28]. In addition, secreted frizzled-related protein-1 (SFRP1), an extracellular antagonist of Wnt, is a cargo protein of Rab37 in cancer cells. SFRP1 is secreted by Rab37 in a GTP-dependent manner to restrain sphere forming ability of lung cancer cells by inhibiting Wnt/β-catenin transactivation [29]. Moreover, we reported a novel regulation of Rab37 activity by PKCα-mediated phosphorylation which inhibits exocytic transport of TIMP1 and thereby enhances lung tumor metastasis [30]. We also discovered that VAMP8 is a novel v-SNARE (vesicle soluble *N*-ethylmaleimide-sensitive factor attachment protein receptor) crucial for Rab37-mediated exocytic transport of TIMP1 to suppress lung tumor metastasis. VAMP8 co-localizes with Rab37 in a GTP-dependent manner and the Rab37-TIMP1 trafficking events are largely reduced in VAMP8 deficient cells [31] (Fig. 2A). More works examining Rab37 and its cargo/effector proteins are underway.

#### Exocytic trafficking of membrane proteins in cancer cells

In the exocytic transport to the PM, Rab8 and Rabin8, a GEF of Rab8, are required for fibroblast growth factor (FGF) 2 secretion to promote the EGF receptor (EGFR) signaling pathway which contributes to lung cancer malignancy [32]. In addition, Rab25 promotes or blocks tumor growth through the chloride intracellular channel protein 3 protein which is necessary for Rab25-mediated integrin recycling from late endosomes/lysosomes and drive cancer progression [33]. Furthermore, Rab8 and Rab2a regulate membrane type 1-matrix metalloproteinase 1 (MT-MMP 1) activity to control exocytosis or degrade ECM, and thus promote invasive breast cancer programs [34, 35] (Fig. 2B). Importantly, exocytotic Rabs can transport vesicles to the cell membrane through the ER-Golgi-PM trafficking pathway for cell membrane fusion and secretion of cargo proteins. These secreted proteins can regulate TME and affect the occurrence and development of tumors [36–38], which is elaborated in the “Recycling-mediated cargo trafficking in cancer cells” section.

### Recycling-mediated cargo trafficking in cancer cells

#### Recycling of integrins in cancer cells

Endocytosis and recycling of key components of ECM and integrin appears to enhance cancer cell adhesion and migration. For example, Rab11a is involved in many cellular functions, such as endosomal membrane organization, cytokinesis and phagocytosis, depending on the interaction with different effectors [39, 40]. For instance, Rab11 interacts with Rab coupling protein (RCP) and acts as a scaffold to promote the interaction of αvβ1-integrin and EGFR1 to enhance cell migration in 3D-culture microenvironments in response to EGF or diacylglycerol kinase α signal [41, 42]. In addition, Rab11-mediated α6β4 integrin trafficking is involved in cancer invasion and metastasis in breast cancer [42, 43]. Notably, Rab25 is presented to the integrin β1-cytotail in its active GTP form, and Rab25 vesicles deliver α5β1 integrin to the PM and localize to the tips of extending pseudopodia during the cell migration within the 3D-culture microenvironments [44]. Rab21 in its GTP-bound form is involved in the recruitment of integrin α-subunits to the endocytic vesicles. The GAP domain of p120RasGAP can interact with cytoplasmic domain of integrin α-subunit and compete with Rab21 for binding to endocytosed integrins [45, 46]. Rab5 mediates vesicle formation and early endosome function by microtubule-dependent adhesion disassembly. Rab5 and its effector protein, Rab interactor 2 (Rin2), recruit R-Ras to the endosomal Rin2–Rab5 complex to further promote internalization of β1-integrin to EEA1-positive early endosome, and subsequently activate Rac via its exchange factor Tiam1, and coordinate endothelial cell adhesion and morphogenesis on fibronectin substrates [47, 48]. The Rab5-mediated recycling of integrins and ECM proteins, with the help of phosphorylated effector Rabaptin-5, is required for efficient cell migration and invasion [49] (Fig. 2C).

#### Recycling of growth factors in cancer cells

Oncogenic Rab35, which was identified by two gain-of-function mutations in tumor cells has been reported to drive the activation of oncogenic PI3K/Akt signaling. The constitutively active Rab35 mediates internalization of platelet-derived growth factor receptor α (PDGFRα) to LAMP2-positive endosomal membrane, where it activates PI3K/Akt signaling in the absence of PDGF ligand [50]. Recently, the comprehensive collection of knock out for the entire Rab family has been done in Madin–Darby canine kidney (MDCK) cells [51, 52]. Of note, the transport of transmembrane cargoes including podocalyxin and transferrin receptor (TfR) from post-Golgi to the PM is mildly delayed in *Rab6*-KO cells [52] (Fig. 2C). Further works are required to identify additional factors or signaling molecules that regulate recycle of growth factor receptors to promote or restrain cancer progression.

### Cooperating Rab-mediated tumorigenesis with cancer cell signaling

Of note, alteration of some Rab proteins impacts tumor progression through affecting signal pathways. For example, Rab23 upregulates MMP9, cyclin E and c-Myc protein expression to promote proliferation and invasion via NF-κB signaling pathway in bladder cancer [53]. Overexpression of Rab25 correlates with aggressiveness of ovarian and breast cancers and promotes invasion and metastasis through upregulating PI3K–AKT pathway [54]. Moreover, colorectal cancer patients with poor prognosis are associated with overexpression of Rab1a, which functions as an mTORC1 activator to enhance mTORC1 signaling and cause tumor progression and invasion [55]. In line with this observation, a recent study showed that Rab3c promotes cell migration and correlates with poor prognosis in colon cancer by regulating the ability of cancer cells to release IL-6 via exocytosis and activating the JAK2-STAT3 pathway [56]. In addition, S-SMAD3, a key modulator in the TGF-β mediated transcriptional activation that drives epithelial–mesenchymal transition (EMT) and cell migration, binds to the promoter of *Rab26* gene, thereby increasing its expression to enhance cancer progression [57]. This is suggestive of the Rab proteins’ role in oncogenic signaling to direct tumor progression.

In contrast to the roles of Rab proteins in promoting tumor progression, a small group of Rab proteins possess the ability to suppress tumor progression. Down regulation of Rab17 induces EMT, enhancing cell proliferation, invasion and migration via STAT3/HIF (hypoxia-inducible factor)-1α/VEGF pathway. Clinical data showed that low Rab17 expression correlates with poor survival in non-small cell lung cancer patients [58]. Rab25 is another Rab that functions as a tumor suppressor in colon cancer as increasing malignant tumor formation in intestinal epithelial cells from the ApcMin/−; Rab25−/− mice are observed [59]. On the other hand, Rab25 mediates both anti-invasive and anti-angiogenic abilities through inhibition of FAK–Raf–MEK1/2–ERK signaling in esophageal squamous cell carcinoma [60]. In addition, Rab25 and Rab 11 work corporately to induce phosphorylation of FAK and promote ovarian cancer cell invasion [61]. Interestingly, Rab37 also exerts tumor suppressive function in cancer epithelial cells (see “Exocytic trafficking of secretory proteins in cancer cells” section), while it functions as a pro-tumor factor in cancer associated immune cells (see “Vesicles trafficking pathways in macrophages” and “Vesicles trafficking pathways in neutrophils” sections). Overall, the relationship between Rab and cancer progression is complex and is dependent on different cell-type specialization and tissue context.

### Rab GTPases regulate the immune-related signaling pathways in TME

**Fig. 3:**
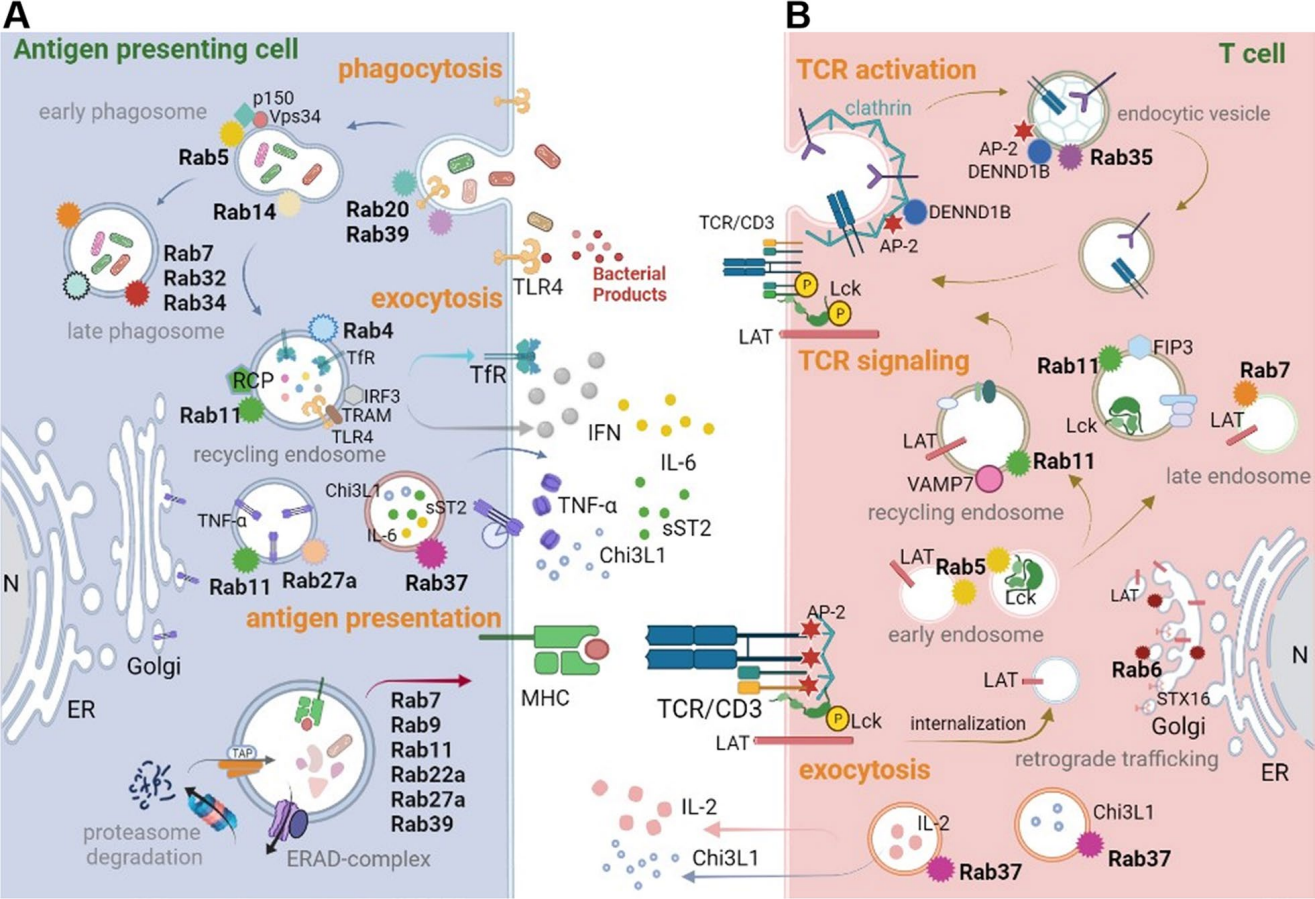
Rab proteins are involved in the crosstalk between antigen presenting cells and T cells in the tumor microenvironment. Aggressive cancer generates an inflammatory TME or a long-term chronic inflammatory response by recruiting infiltrated immune cells leading to immune cell inactivation and cancer cell proliferation, further enabling mutual cell–cell interaction through direct and indirect crosstalk. The direct communication includes the membrane receptor/ligand traffic, while indirect routes are mediated by soluble molecules from lytic pathogen, cytokines and chemokines. The Rab proteins-mediated crosstalk in **A** antigen presenting cells, commonly macrophages and DCs and in **B** adaptive immune T cells. Together, these Rab proteins regulate stepwise changes in cellular interaction partners to foster an immunosuppressive TME to promote cancer progression

Immune cells in TME regulate tumor growth through innate and adaptive immune responses. Myeloid cells in the innate immunity provide a first line of defense against many common pathogens and are critical for the control of bacteria or virus infections. The innate immune response is vital for the activation of adaptive immunity [62, 63]. Intracellular trafficking must be tightly regulated in immune cells. We will describe recent advances of Rab-mediated conventional protein secretion (CPS) in immune cells in TME in this section (Fig. 3).

#### Vesicles trafficking pathways in macrophages

Pathogen recognition is an important part of the innate response, and phagocytosis is involved in clearance of pathogens or dead cells. The expression of pathogen associated molecular patterns (PAMPs) [64] is recognized by pattern recognition receptors (PRRs), including mannose-receptor, Dectin-1, scavenger receptor A, CD14 and CD36 on the cell membrane [65] and thereby induces downstream immune regulatory signaling and functions [64, 66, 67]. PRRs trafficking to cell membrane and secretion of immune modulators in the microenvironment require the Rab proteins. For example, in phagosome maturation, Rab5 and Rab14 localized on early phagosomes generated by the fusion of nascent phagosome with early endosome [65, 68]. Rab5 is activated by the regulator GAP and vacuolar protein sorting 9 (VPS9) domain containing protein 1 (GAPVD1). Rab5 is important for phagosome development, as disruption of its function can arrest maturation, precluding the formation of late phagolysosomes and phagolysosomes. Rab5 recruits and activates multiple effector proteins such as EEA1, p150–VPS34 complex and Mon1a/b. The p150 is VPS15-like serine/threonine kinase. VPS34 is involved in vesicle-mediated vacuolar protein sorting and belongs to class III PI3K. The p150–VPS34 complex binds directly to active Rab5, then Rab5 drives its catalytic activity to synthesize PI(3)P locally and accumulation of this signaling phosphoinositide leads to subsequent steps in phagosome maturation [65]. However, certain compartments of the early phagosome, such as TfR, need to be recycled back to the PM. Therefore, RCP interacts with Rab4 and Rab11 at the endosomal compartment and regulates the trafficking of TfR back to the PM [69]. Multiple Rabs are known to function in phagosome maturation. For example, Rab20 and Rab39 regulate the phagosomal acidification, while Rab20, Rab22b, Rab32, Rab34, Rab38 and Rab43 modulate the recruitment of cathepsin D to the phagosome [70, 71] (phagocytosis, Fig. 3A).

Rab-mediated vesicle trafficking is also important to immune signaling and cytokine/chemokine secretion. Rab11 mediates the delivery of the Toll-like receptor 4 (TLR4) from endocytic recycling compartments to phagosome. Furthermore, TIR-domain-containing adapter-inducing interferon (IFN)-β (TRIF) is an important adaptor protein responding to activation of TLRs [72]. Rab11 regulates the TRIF-related adaptor molecular (TRAM) recruitment into phagosomes and promotes IFN regulatory transcription factor 3 (IRF3) signaling pathway leading to the type I IFNs secretion [73]. Rab11 also contributes to TNF-α delivery in macrophages, however, inactive Rab11 blocks newly synthesized TNF-α in Golgi, suggesting that Rab11 is a key player in the secretion pathway of TNF-α [74] (exocytosis, Fig. 3A).

Notably, Rab37 regulates the exocytosis of soluble ST2 (sST2) to extracellular compartment to act as a decoy receptor to interrupt IL-33/ST2L signaling in macrophages and mediates macrophage polarization to the M1-like phenotype in TME of lung cancer [8]. Rab37 also mediates IL-6 secretion in macrophages. The secreted IL-6 acts in a paracrine manner to promote STAT3-dependent *PD-1* mRNA expression in CD8^+^ T cell to foster an immunosuppressive TME [6]. Moreover, Rab37 mediates intracellular vesicle trafficking and exocytosis of chitinase 3-like-1 (CHI3L1), a protumor secretion glycoprotein associated with the immunosuppressive TME, in a GTP-dependent manner which is abolished in the splenocytes and macrophages from *Rab37* KO mice. The secreted CHI3L1 activates AKT, β-catenin and NF-κB signal pathways in cancer cells and elicits a protumor TME characterized by activating M2 macrophages and increasing the population of regulatory T cells [75] (exocytosis, Fig. 3A). In view of the last-mentioned three studies, it is interesting to identify additional cargoes of Rab37 in tumor associated macrophages in TME.

#### Vesicles trafficking pathways in neutrophils

Neutrophils play a vital role in the innate immunity. The recruitment of neutrophils from the intravascular compartment into infected or injured tissue is an essential component of the inflammatory response [76]. The contribution of Rab proteins in neutrophil functions has been noticed. For example, Rab27a plays a critical role in neutrophil infiltration and degranulation. Rab27a interacts with Munc13-4, a member of the Munc13 family of proteins involved in vesicle priming function, for MMP9 containing gelatinase granule released, while it interacts with Munc13-4 and synaptotagmin-like protein1 (Slp1) for plasma myeloperoxidase inflammatory factor and neutrophil elastase serine protease-containing azurophilic granules released [77]. Notably, Rab27a upregulates response to inflammatory stimulation by enhancing exocytosis of TNF-α [78], whereas downregulation of Rab27a correlates with lower neutrophil-mediated tumor cytotoxicity in TME [79, 80] (exocytosis, Fig. 3A).

In addition, polarized vesicle transport is a key player in neutrophil polarization. The protein kinase PKN1 promotes the polarized Rab21 via phosphorylation of Rabphilin-3A, an essential protein in docking and fusion steps of regulated exocytosis in neutrophils. Subsequently, polarization of PIP5K1C90, a plasma membrane associated lipid kinase that interacts with talin and endocytic structural protein adaptin-2 (AP2), mediates the synthesis of phosphatidylinositol 4,5-bisphosphate (PtdIns4,5P2) phospholipid component to mark the specific plasma membrane domain. This process can regulate RhoA activation and increase integrin affinity and neutrophil adhesion to endothelial cells [81]. However, the direct role of Rab-mediated exocytosis in neutrophils in TME remains elusive.

#### Vesicles trafficking pathways in antigen presentation

Many studies in the past have shown that Rab proteins are involved in the inflammatory response of immune cells [82, 83], speculating that vesicle trafficking plays roles in TME preceded or accompanied by chronic inflammation [84, 85]. In the initiation of cytotoxic immune response, the activated T cells recognize the matched antigen on the surface of antigen-presenting cells (APCs) such as macrophages and dendritic cells (DCs). In adaptive immune responses, Rab GTPases are key regulators in membrane trafficking of major histocompatibility complex (MHC) molecules. Intracellular transfer of MHC–peptide complexes in APCs is a continuous process that begins from exogenous antigen internalization and ends with the vesicle trafficking and presentation of MHC-peptide complexes on the cell surface [86, 87]. In general, MHC class I and class II molecules present peptides from different sources. Antigen degraded by the proteasome in the cytosol is transported to the ER, where the digested peptides interact with the associated transporters which load the antigen peptides onto the MHC-I molecules. Once MHC-I molecules receive peptides in the ER, the peptide/MHC-I complex are transported along the secretory pathway to the cell surface for presentation to CD8^+^ T cells. In contrast, vesicles carrying with MHC-II molecules are able to fuse with endosomes or phagosomes to load peptides on MHC-II, thereby the peptide/MHC-II complex is delivered to cell surface for recognition by CD4^+^ T cells [88].

To generate efficient T cell response, it is important for antigen processing to avoid complete degradation of the antigen, thus, Rab GTPase dependent trafficking contributes to slow down the vesicle acidification and phagosome maturation in DCs. A proteomic analysis with purified phagosomes was conducted to search for molecules involved in the regulation of phagosomal function in DCs. Among them, Rab27a involved in the regulation of the motility and exocytosis of secretory granules is revealed as an important regulator. Rab27a mediates the recruitment of NADPH oxidase NOX2, which maintains the phagosomal pH value to prevent over-acidification and antigen degradation in the phagosome [89]. Furthermore, Rab22a and Rab39 also increase the levels of Rab GEF Sec22B and NOX2 to facilitate the delivery of MHC-I molecules from the ER to the phagosome and bind to specific peptides for transport and presentation to cell surface of CD8^+^ T cells [90, 91] (antigen presentation, Fig. 3A).

#### Vesicles trafficking pathways in T cells

Of note, Rab35 and its GEF, DENND1B, are critical for negative regulation of T cell receptor (TCR) signal in Th2 cells. Knockdown of Rab35 activates the TCR signal and increases cytokine production of IL-4, IL-5 and IL-13 in Th2 cells. However, TCR-mediated signaling pathway is promoted when DENND1B interacts with Rab35 and clathrin adaptor AP-2 to recycle TCR/CD3 to the PM in Th1 cells. Together, these results indicate that Rab35 downmodulates TCR signaling in Th2 cells, while upregulates TCR signaling in Th1 cells [92, 93] (TCR activation, Fig. 3B).

On the other hand, Rab11 and its effector, Rab11 family interacting protein-3 (Rab11-FIP3), regulate the expression of TCR/CD3 on cell surface through the modulation of steady-state Lck-mediated TCRζ phosphorylation and control TCR signal transduction and T cell activation [94]. In addition, T cell activation via TCR signaling pathway leads to the recruitment and activation of downstream signaling molecules, such as linker for activated T cells (LAT), the adaptor protein which mediates the T cell activation. It is found that LAT containing vesicles co-localize with late endosome marker Rab7 [95]. Moreover, the retrograde trafficking of LAT internalized from PM to Golgi/TGN regulates the polarized delivery of LAT that is dependent on the Rab6 and the t-SNARE Syntaxin-16 (STX16) [96] (TCR signaling, Fig. 3B).

Importantly, Rab37 colocalizes with IL-2 at the immune synapse, while it colocalizes with TNF in the cytoplasmic compartment in Th1 cells [97]. Of note, Rab37 has recently shown to regulate exocytotic secretion of pro-tumor glycoprotein CHI3L1 from T cell in a GTP-dependent manner to activate M2 macrophages and increase the population of regulatory T cells via activating AKT, β-catenin and NF-kB signal pathways in TME of lung, pancreatic and colon cancers [75] (exocytosis, Fig. 3B).

Overall, the expression of Rab GTPases is tightly regulated by cytokines in the microenvironment, and Rab protein networks are associated with various inflammatory processes. In the future, it is critical to further define the molecular basis of Rab regulation in conventional vesicular transport during tumorigenesis and its role in tumoral immunity.

## Mechanisms of extracellular vesicles associated with communication between tumor and stromal cells

### Subtypes, biogenesis and secretion of EVs

#### Main subpopulations of EVs

**Fig. 4:**
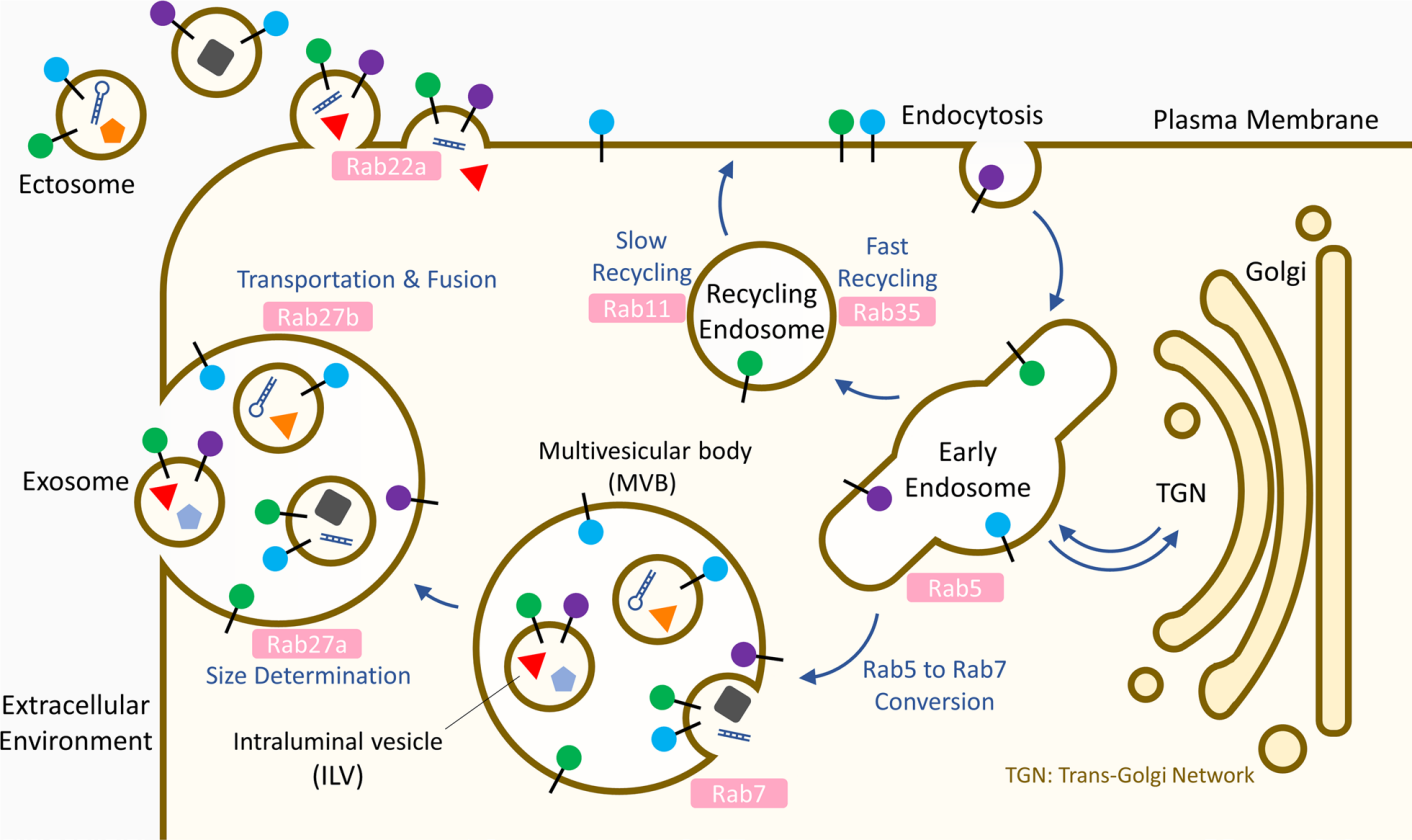
Intracellular trafficking routes in the biogenesis of exosomes and ectosomes. During the biogenesis of exosomes, Rab GTPases play an important role in regulation of multiple intracellular trafficking steps including cargo collection into MVBs, transportation of MVBs toward plasma membrane and membrane fusion for ILVs release. Besides, the Rab family is also involved in ectosome formation

EVs, naturally released by almost all cells, comprise of a wide variety of lipid bilayer vesicles. According to their biogenesis, release pathways, size, content and function, three main subtypes of EVs have been identified and classified as ectosomes (also called microparticles and microvesicles) (100–1000 nm), exosomes (30–150 nm) and apoptotic bodies (50–5000 nm) [98] (Fig. 4). Apoptotic bodies are released as a product of apoptotic cell disassembly in a manner that promotes their clearance by phagocytes to avoid inflammation [99]. Based on the phosphatidylserine externalization, annexin V is a commonly used marker for labeling apoptotic bodies [100]. Apart from apoptotic bodies, both ectosomes (bearing CD9 and CD81) and exosomes (bearing CD9, CD63 and CD81) carry a selected profile of proteins, lipids, and nucleic acids from the donor cells and thus deliver a complex array of information to neighbor or distant recipient cells [101, 102]. Specifically, ectosomes are secreted by directly shedding or outward budding of the PM, while exosomes originate from inward budding of early endosomal membrane resulting in the progressive accumulation of intraluminal vesicles inside MVBs that can be released by exocytosis pathway [103]. However, since part of ectosomes are of similar size as exosomes, and substantial overlap of cargoes is often observed, it remains extraordinarily difficult to distinguish the two types of EVs by simple isolation using ultracentrifugation without tracking their formation process by imaging technologies. In the present review, we will focus on ectosomes and exosomes to summarize the available information regarding the functions and roles of EVs in intercellular communication within TME.

#### Biogenesis and secretion of EVs

Exosomes are originally formed as intraluminal vesicles (ILVs) accumulated within MVBs. During the formation of ILVs, the endosome sorting complexes required for transport (ESCRT) machinery functions as the key mediator in cargo sorting and vesicle budding [104]. The ESCRT mechanism is initiated by ESCRT-0, which recognizes and clusters ubiquitylated cargoes on microdomains of endosomal membrane [105]. Next, ESCRT-0 recruits ESCRT-I which interacts and cooperates with ESCRT-II to facilitate endosomal membrane invagination [104, 106, 107]. Further, ESCRT-II recruits ESCRT-III, which cleaves the buds to generate ILVs and then leaves for additional rounds of budding [108]. Cargo sorting may also occur by syndecan (SYN), syntenin (STN) and the ESCRT accessory protein ALG-2 interacting protein X (ALIX), which connect the cargoes to ESCRT-III for ILV biogenesis [109].

Although ESCRT machinery is predominantly involved in cargo packing, exosomes can also be formed through ESCRT-independent pathways. Notably, several tetraspanins (CD9, CD63, CD81 and CD82), the most used exosome markers with partially characterized function in exosomes, have recently been discovered to be directly involved in cargo selection and membrane structure remodeling [110]. Mechanically, these tetraspanins form clusters to interact with sorting target receptors and signaling molecules on the microdomains of membrane, and then the cone-shaped clustering induces inward curvature of the endosomal membrane [111–113]. In addition to tetraspanin-mediated exosome biogenesis, ceramide, a bioactive lipid produced during the hydrolysis of sphingomyelin by neutral sphingomyelinase 2 (n-Smase2), was reported to trigger membrane curvature and ILV formation [114]. Indeed, the mechanism of exosome biogenesis is currently far from being comprehensively understood. It is suggested that ESCRT-dependent and ESCRT-independent mechanisms may operate differently depending on the cell type and the signaling stimuli.

Rab GTPases that mediate intracellular vesicle trafficking are also essential players for exosome production (Fig. 4). The first Rab GTPase discovered to regulate exosome secretion is Rab11 [115], which is involved in slow recycling route of exosomal pathway in a Ca^2+^-dependent manner [116, 117]. Besides, Rab35, a master regulator of fast recycling route, is also engaged in exosome secretion [118]. Of note, Rab27a and Rab27b are important in regulation of MVBs toward PM for docking and fusion [119]. Using total internal reflection fluorescence (TIRF) microscopy, it was found that Rab27a is critical for size determination of MVBs, and Rab27b transfers MVBs from microtubules to the actin-rich cortex at the cell periphery [119]. Previous studies showed that Rab11-, Rab35- and Rab27b-regulated MVBs co-localized with synaptosomal-associated protein 23 (SNAP23), a vital SNARE protein regulating the docking and fusion of transport vesicles with PM, confirming the release of Rab-mediated exosome through PM via the SNARE machinery [120, 121]. Importantly, recent studies reported that the expression of both Rab27a and Rab27b can be upregulated under hypoxia condition through direct transcriptional regulation by HIF-1α [122] and HIF-2α [123], respectively, revealing the significance of Rab27a and Rab27b in biogenesis of EVs within TME. In addition, the Rab5–Rab7 conversion, which regulates early-to-late endosome transition, contributes to the transfer of cargoes to MVBs [124, 125]. Interestingly, in addition to Rab27a and Rab27b, the small hairpin RNA (shRNA)-based screening targeting 59 Rab GTPases revealed that Rab2b, Rab5a and Rab9a are also required for exosome secretion in HeLa cells [119]; however, the functions of Rab2b and Rab9a in exosome trafficking remain unclear. Many studies are underway to explore and better understand the coordination of exosome trafficking by Rab GTPases family.

The molecular mechanisms of ectosome biogenesis are far less characterized as compared to exosome. Nevertheless, an ESCRT-I subunit tumorsusceptibility 101 (TSG101) has been observed to be recruited to the PM, leading to the release of ectosome [126]. Additionally, enzymatic machineries, which induce physical bending of the membrane and restructuring of the underlying actin cytoskeleton, are also required for ectosome generation. Of note, the n-Smase2/ceramide machinery that triggers ILV formation can also promote membrane bending for ectosome shedding [127]. In addition, it was reported that phospholipid transportation enzymes (flippases, floppases and scramblases) induce membrane budding and formation of ectosomes via driving the collapse of the asymmetric phospholipid distribution [128]. Besides, the cytoskeletal element (myosin-1a) and cytoskeletal regulator (RhoA) cause mechanical force on PM, leading to the formation and release of ectosomes [129, 130]. Remarkably, Rab22a, a novel downstream transcriptional target of HIF-1, was found to be indispensable for the formation of hypoxia-induced ectosome in breast cancer [131]. In addition, the production of triple-negative breast cancer-derived ectosomes along with the growth and metastasis of cancer cells were significantly suppressed after Rab22a inhibition [132], highlighting the anti-cancer therapeutic potential of targeting ectosome formation. Indeed, as we are only beginning to explore the ectosome field, further studies are urgently needed for complete understanding of ectosome biology.

### Functions of EVs in TME

During tumor progression, the reciprocal communication between cancer cells and their microenvironment, including immune cells, stromal cells and ECM, forces these immune and stromal cells to acquire a pro-tumor phenotype, leading to accelerated development and invasion of tumor cells [133, 134]. Secreted EVs, in addition to chemokines and cytokines, have been recognized as crucial mediators of information exchange to regulate local and distant TME. We have summarized the EVs and their cargoes affecting TME in this section (Table 1).

**Table 1:** Functions of EVs and their cargoes released by different cells in TME

Donor of EVs	Cargoes	Recipients	Responses of recipients	References
Cancer cells	Mutant KRAS	Cancer cells	Aggressive**↑**	[135]
	EGFRvIII	Cancer cells	Drug resistance**↑**	[136]
	MDR1	Cancer cells	Drug resistance**↑**	[137]
	HSP70	NK cells	Cytolytic activity**↑**	[138]
	Tumor antigens	DCs	Cross-presentation**↑**	[139]
	PD-L1	CD8 T cells	Apoptosis**↑**	[143, 144]
	FasL	CD8 T cells	Apoptosis**↑**	[145]
	TRAIL	CD8 T cells	Apoptosis**↑**	[146]
	NKG2D	CD8 T cells, NK cells	Cytotoxicity**↓**	[147]
	HSP72	MDSCs	Immunosuppression**↑**	[148]
	miRNAs, lncRNAs	M2 macrophages	Immunosuppression**↑**	[149]
	TGF-β1	Treg	Treg differentiation**↑**	[150]
	TGF-β1	Fibroblasts	CAF activation**↑**	[151]
DCs	TNF, FasL, TRAIL	NK cells	IFNγ secretion**↑**	[153]
	Tumor antigens	B cells	Proliferation IgG production**↑**	[154]
	Tumor antigens	CD8 T cells	CD8 T cell activation**↑**	[154]
NK cells	Perforin, granulysin, granzyme	Cancer cells	Apoptosis**↑**	[155]
γδ T cells	NKG2D, FasL, TNFα, IFNγ, perforin	Cancer cells	Tumor growth**↓**	[156]
M1 macrophages	M1 marker mRNA	M2 macrophages	M2 → M1 Repolarization**↑**	[157]
	Tumor antigens	CD8 T cells	CD8 T cell activation**↑**	[158]
M2 macrophages	miRNAs	Cancer cells	Tumor progression**↑**	[160]
MDSCs	miRNAs	Cancer cells	Tumor growth**↑**	[161]
	TNF⍺, FasL	CD8 T cells	Activation-induced cell death (AICD)**↑**	[164]
	CD47, TSP1, S100A8/9	MDSCs	MDSCs Recruitment**↑**	[165, 166]
Treg	CD73	CD8 T cells	CD8 T cell activation**↓**	[167]
B cells	CD73	CD8 T cells	CD8 T cell activation**↓**	[122]
CAFs	SHH	Cancer cells	Aggressive**↑**	[169]
	miRNAs	Cancer cells	Proliferation and metastasis**↑**	[170]
	miRNAs	Cancer cells	Chemo- or radio-resistance**↑**	[171–173]
	metabolites	Cancer cells	Metabolic reprogramming**↑**	[174]
	circRNA	Cancer cells	PD-L1 expression**↑**	[175]
MSCs	miRNAs	Cancer cells	Proliferation**↑**	[178]
	miRNAs	M2 macrophages	M2 polarization**↑**	[178]
	PD-L1, CD73, TGF-β1	CD8 T cells	Immunosuppression**↑**	[179]

#### Cancer cell-derived EVs in TME

Many studies have shown compelling evidence that cancer-derived EVs carry various signaling molecules to promote tumor progression. By proteomic analysis, mutant KRAS colon cancer-derived EVs were discovered to transport mutant KRAS and other oncogenic proteins such as EGFR, SRC family kinases and integrins, toward neighbor cancer cells with wild-type KRAS, leading to enhanced growth of these neighbor cancer cells [135]. Besides, the oncogenic form of EGFR, known as EGFRvIII, can be shared between glioma cells via intercellular transfer of EVs, resulting in reprogramming of recipient cells toward oncogenic EGFRvIII-dependent phenotype [136]. Furthermore, docetaxel-resistant cancer cell-secreted EVs can deliver multidrug resistance protein 1 (MDR1), enabling recipient chemosensitive cells to develop new acquired drug resistance [137] (Table 1).

Importantly, cancer-derived EVs also induce immunostimulatory or immunosuppressive effects in TME. It was demonstrated that cancer cell secreted heat shock protein 70 (HSP70)-loaded EVs could boost migration and granzyme B-dependent cytolytic activity of natural killer (NK) cells [138]. Besides, tumor-derived EVs were discovered to carry multiple tumor antigens, and these antigens were uptaken by DCs and cross-presented to cytotoxic T lymphocytes (CTLs), promoting activation of CTL responses against tumors in vivo [139]. Notably, there is no report about direct activation of T cell by cancer-derived EVs, suggesting that cancer-derived EVs-induced CTL activation requires antigen processing and presentation by APCs [140–142]. On the contrary, cancer-derived EVs are enriched in immunosuppressive proteins, including death receptor ligands such as PD-L1 [143, 144], FasL [145] and TRAIL [146], by which cancer-derived EVs directly induce apoptosis of anti-tumor T cells. In particular, the pre-treatment level of circulating exosomal PD-L1 are significantly higher in melanoma patients who have poor responses to anti-PD1 therapy [143], revealing that the level of circulating exosomal PD-L1 may help predict anti-PD1 response and clinical outcomes. Besides, cancer-derived EVs also express ligands for NKG2D, an activating receptor mediating killing effects of NK cells and CTLs, and thus impair cytotoxicity of NK and CD8^+^ T cells [147] (Table 1).

Moreover, cancer-derived EVs can transport various cargoes for broad enhancement of immune suppression, such as heat shock protein 72 (HSP72) for promoting suppressive functions of myeloid-derived suppressor cells (MDSCs) [148], miRNAs and lncRNAs for boosting activation of M2-like pro-tumor macrophages [149], and TGF-β1 for augmenting differentiation of regulatory T cells (Treg) [150]. Of note, TGF-β1 delivered by cancer-derived EVs also triggers the activation of stromal fibroblasts into cancer-associated fibroblasts (CAFs) [151], which regulate ECM remodeling to create a TME favorable for tumor growth and metastasis (Table 1, also see “Stroma cell-derived EVs in TME” section).

To date, numerous efforts in exploring EVs secreted by cancer cells and their bioactive cargoes are still underway to gain a comprehensive understanding of their roles in the molecular symphony of TME. Based on current published evidence, it is likely that the dual roles of cancer-derived EVs in immunomodulation are dynamically altered during tumor progression. Namely, early-stage tumor-secreted EVs may transport few immunosuppressive cargoes along with tumor antigens to stimulate immune response, while late-stage tumor-released EVs carry abundant immunosuppressive cargoes, such as TGF-β1 and PD-L1. Further exploring of cancer-derived EVs is important to elucidate their dual role in TME during tumor progression.

#### Immune cell-derived EVs in TME

During tumor progression, tumor tissues are commonly infiltrated by immune cells, including DCs, macrophages, NK cells, T and B lymphocytes, and neutrophils. Growing evidence shows that the dynamic reciprocal communications by transferring EVs between cancer and tumor-infiltrating immune cells (TIICs) is critical in determining tumor fate [152]. Indeed, TIIC-derived EVs play divergent roles in cancer immunity. It was reported that EVs derived from DCs carry TNF, FasL and TRAIL. The DC-derived EVs can not only induce apoptosis of cancer cells but also promote NK cells to secrete IFN-γ [153]. Additionally, EVs from antigen-pulsed DCs significantly increase germinal center B cell proportions and elicit antigen-specific IgG production as well as CD8 T cell activation [154]. Besides, NK cells can secrete EVs containing high level of perforin, granulysin and granzymes A and B, thus triggering caspase pathways in targeted cancer cells [155]. Notably, EVs derived from γδ T cells, a unique population of T cells sensing target antigens in an MHC-independent way, express NKG2D, FasL, TNFα, IFN-γ and perforin on their membrane surface and are able to significantly reduce tumor growth [156]. Interestingly, M1-like anti-tumor macrophage-derived EVs can transport higher mRNA amounts of M1 markers, including CD86, IL-6, TNF-α and iNOS, toward M2 macrophages and further induce M2 to M1 repolarization [157]. Interestingly, a recent study mimicked the phagocytosis of tumor cell nuclei by M1 macrophages, and these hybrid macrophages secreted so-called chimeric exosomes, which expressed tumor antigens to promote T cell activation, thus leading to inhibition of tumor progression and protection from tumor recurrence [158] (Table 1).

Nevertheless, hypoxic tumor cells release immunosuppressive EVs to amplify polarization and recruitment of M2 macrophages [159]. Reciprocally, M2 macrophages can deliver miRNA-containing EVs to cancer cells to promote tumor progression [160]. Besides, MDSCs secrete EVs loaded with miR-143-3p to activate PI3K/Akt signaling of cancer cells and thus accelerate tumor growth [161]. Additionally, abnormal accumulation of MDSCs and their EVs also contributes to shaping immunosuppressive TME [162, 163]. A recent study demonstrates that MDSC-derived EVs loaded with TNFα and FasL simultaneously enhance IFN-γ production and trigger Fas/FasL pathway in T cells, leading to hyper-activation and activation-induced cell death (AICD) of T cells [164]. Particularly, MDSC-derived EVs are enriched in chemotactic proteins, such as S100A8/9, CD47 and TSP1, thus facilitating recruitment of MDSCs to the tumor tissue and pre-metastatic niche [165, 166]. In addition, it was found that Treg-derived EVs express CD73 to convert extracellular adenosine-5-monophosphate to adenosine [167], a well-known modulator suppressing the activation of naïve CD8 T cells by inhibiting TCR signaling. Interestingly, B cell-derived EVs also contain CD73 and regulate ATP-adenosine metabolic process, and thereby inhibiting CD8 T cell proliferation [122] (Table 1).

Taken together, TIIC-derived EVs can be regarded as miniatures of donor cells due to the same lipid membrane structure and similar bioactive molecular profiles. Exploring the dynamically altering fraction of TIIC-derived EVs in the total EV population is of great significance for understanding the overall immunomodulation of TME during tumor progression.

#### Stroma cell-derived EVs in TME

In addition to immune stromal cells, CAFs constitute the most prominent component of tumor stroma. Particularly, CAFs are often found to have pro-tumorigenic properties by secreting growth factors, cytokines and ECM molecules [168]. Besides, CAFs also secrete EVs to communicate with cancer and immune cells during tumor progression. Recently, CAF-derived EVs contain a high level of stemness-related protein Sonic Hedgehog (SHH), which is subsequently taken up by esophageal squamous cell carcinoma and enhance their aggressiveness [169]. In addition, CAF-derived EVs was discovered to transfer miR-500a-5p, by which ubiquitin-specific peptidase 28 (USP28) was directly targeted and blocked in breast cancer cells, thus promoting proliferation and metastasis of cancer cells [170]. Furthermore, several miRNAs transferred by CAF-derived EVs can also confer chemotherapeutic or radiotherapeutic resistance in cancer cells [171–173]. Of note, CAF-derived EVs induce the switch of cancer cells toward Warburg phenotype [174], allowing rapid biosynthesis of ATP. Remarkably, these CAF-derived EVs contain intact metabolites, including amino acids, lipids and TCA-cycle intermediates, which can fuel metabolites activity to promote tumor growth under nutrient stress conditions [174]. Interestingly, CAFs secrete circEIF3K-loaded EVs under hypoxia treatment, and circEIF3K in these EVs enhance the expression of PD-L1 in colorectal cancer cells [175]. Although the effects of CAF-derived EVs on immune cells remain largely unknown and are being actively investigated, these EVs were noticed to carry large amounts of TGF-β1 [176], suggesting their involvement in shaping an immunosuppressive TME (Table 1).

Besides, mesenchymal stem cells (MSCs) are multipotent stromal cells that can differentiate into various types of cells. Growing evidence showed that the crosstalk between MSCs and other cells within TME plays a crucial role in tumor progress ion [177]. Notably, MSC-derived EVs was shown to carry miR-21-5p, which not only facilitated lung cancer cell proliferation, intra-tumoral angiogenesis and tumor growth, but also promoted M2 macrophage polarization [178]. Furthermore, by proteomic analysis, PD-L1, CD73 and TGF-β1 were revealed as bioactive cargoes of MSC-derived EVs, suggesting the immunosuppressive effects of MSC-derived EVs on T cells [179] (Table 1).

Still, the specific contribution of stromal cell-derived EVs to immunomodulation of TME and tumor progression remains largely unexplored. Therefore, further studies elucidating the bioactive cargoes of stromal cell-derived EVs and the molecular mechanism underlying intercellular communication within TME would provide insight into the development of anti-tumor therapeutic application.

### Diagnostic and therapeutic applications of EVs

Of great importance, pre-clinical studies showed that EVs and their cargoes could be utilized as biomarkers for cancer early diagnosis, prognosis prediction and therapeutic efficacy evaluation [180, 181]. In particular, a recent clinical trial (ClinicalTrials.gov identifier: NCT02862470) reported that thyroglobulin in urine EVs can be a novel biomarker to predict the prognosis or recurrence of patients with thyroid cancer [182]. Strikingly, another clinical trial (ClinicalTrials.gov identifier: NCT02702856) that recruited 2000 patients showed that a novel urine exosome gene expression assay is superior to the standard of care for predicting high-grade prostate cancer [183]. Although there are still limitations in using EVs as a source of tumor biomarkers, the advantages of liquid biopsy make EVs a potential and powerful approach for clinical diagnosis [184] (Table 2).

**Table 2:** Clinical trials of EVs for development of diagnostic biomarkers

Cancer type	Title of the trial	State	Number of participants	ClinicalTrials.gov Identifier
Thyroid cancer	Anaplastic thyroid cancer and follicular thyroid cancer-derived exosomal analysis via treatment of lovastatin and vildagliptin and pilot prognostic study via urine exosomal biological markers in thyroid cancer patients	Completed	22	NCT02862470 [182]
Lung cancer	Serum exosomal long noncoding RNAs as potential biomarkers for lung cancer diagnosis	Completed	1000	NCT03830619
Pancreatic cancer	Diagnostic accuracy of circulating tumor cells (CTCs) and onco-exosome quantification in the diagnosis of pancreatic cancer—PANC-CTC	Completed	52	NCT03032913
	Acquisition of portal venous circulating tumor cells and exosomes from patients with pancreatic cancer by endoscopic ultrasound: a prospective study	Ongoing	Estimated 30	NCT03821909
Prostate cancer	Clinical validation of a urinary exosome gene signature in men presenting for suspicion of prostate cancer	Completed	2000	NCT02702856 [183]
	Clinical evaluation of ExoDx™ prostate (IntelliScore) in men presenting for initial prostate biopsy	Completed	120	NCT04720599
Rectal cancer	Exosomal as correlative biomarker in clinical outcomes in patients undergoing neoadjuvant chemoradiation therapy for rectal cancer	Ongoing	Estimated 30	NCT03874559
	A prospective, observational, multicenter study on biomarkers for predicting the efficacy and toxicities of neoadjuvant chemoradiotherapy for locally advanced rectal cancer based on tissue and plasma exosome RNA	Ongoing	Estimated 250	NCT04227886
Gastric cancer	Circulating exosomes as potential prognostic and predictive biomarkers in advanced gastric cancer patients: a prospective observational study (“EXO-PPP Study”)	Ongoing	Estimated 80	NCT01779583
Ovarian cancer	Non-coding RNA in the exosome of the epithelia ovarian cancer	Ongoing	Estimated 160	NCT03738319
Bladder cancer	A study of circulating exosome proteomics in gallbladder carcinoma patients	Ongoing	Estimated 50	NCT03581435
Cholangiocarcinoma	Exosomes-derived ncRNAs as biomarkers in cholangiocarcinoma patients	Ongoing	Estimated 80	NCT03102268

In addition, based on their natural advantages, EVs serve as promising carriers for the delivery of therapeutic agents [185]. Recently, the treatment of clinical-grade GMP engineered MSC-derived EVs has been developed to deliver siRNA for targeting oncogenic KRAS, and the treatment of these EVs inhibits tumor growth of pancreatic xenografts and prolongs survival of mice [186], and this treatment is now undergoing clinical trials (ClinicalTrials.gov identifier: NCT03608631). Besides, a phase II clinical trial (ClinicalTrials.gov identifier: NCT01159288) launched for advanced non-small cell lung cancer demonstrated that DC-derived EVs containing tumor-associated antigen can boost NK cell effector function and improve patient survival [187]. Importantly, the therapeutic potential of EVs is reflected by their application in a growing number of clinical trials. Therefore, the expanding knowledge about the biological effects of EVs would provide more insight into the development of therapeutic applications (Table 3).

**Table 3:** Clinical trials of EVs for development of anticancer treatment

Cancer type	Title of the trial	Phase	State	Number of participants	ClinicalTrials.gov identifier
Lung Cancer	Phase II trial of a vaccination with tumor antigen-loaded dendritic cell-derived exosomes on patients with unresectable non-small cell lung cancer responding to induction chemotherapy	II	Completed	41	NCT01159288 [187]
Pancreatic Cancer	Phase I study of mesenchymal stromal cells-derived exosomes with KrasG12D siRNA for metastatic pancreas cancer patients harboring KrasG12D mutation	I	Ongoing	Recruiting	NCT03608631 [186]
Colon Cancer	Phase I clinical trial investigating the ability of plant exosomes to deliver curcumin to normal and malignant colon tissue	I	Ongoing	Recruiting	NCT01294072
Head and Neck Cancer	Preliminary clinical trial investigating the ability of plant exosomes to abrogate oral mucositis induced by combined chemotherapy and radiation in head and neck cancer patients	I	Ongoing	60	NCT01668849

## Mechanisms of autophagy-mediated communication between tumor and stromal cells

### Biogenesis of secretory autophagy

Proteins lacking signal peptides are destined to undergo unconventional secretory routes instead of the conventional ER-Golgi route. According to recent classification, there are four types of unconventional protein secretion (UCPS). Type I UCPS is characterized as a cellular protein releasing via pore-mediated translocation across the PM. For example, when FGF2 binds phosphatidylinositol 4,5-bisphosphate (PtdIns4,5P2) on the cytoplasmic leaflet of the PM, it subsequently promotes phosphorylation of FGF2 on Y82 by Tec kinase [188]. PtdIns4,5P2 then induces self-oligomerization at the PM, which in turn facilitates the formation of lipid membrane pore to allow the translocation of FGF2 to extracellular membrane [189]. On the other hand, type II UCPS requires ABC transporters to help protein translocation across the PM. This type of UCPS has been shown to help the secretion of acylated peptides and yeast mating peptides [190, 191]. Type III UCPS is autophagosome/endosome-based secretion pathway. Proteins via type III UCPS rely on membrane-bound intermediates for secretion. Type IV UCPS is closed to the conventional ER-Golgi route, but it bypasses Golgi to help protein secretion. Proteins carrying signal peptides or transmembrane domains enter the ER but bypass Golgi apparatus and then reach the PM for secretion [192]. Cystic fibrosis transmembrane conductance regulator (CFTR) has been revealed to be secreted by this Golgi bypass mechanism [193, 194]. Among four types of unconventional protein secretion, type III UCPS shows that deprivation of autophagy-related genes can attenuate the secretion of protein to extracellular space. Accordingly, this process is also termed as secretory autophagy.

A complete process of autophagy is referred to autophagic flux which consists of four main steps: (I) autophagosome initiation, (II) autophagosome nucleation, (III) autophagosome elongation and (IV) autophagosome completion and fusion with lysosome. In brief, activation of ULK1/2 kinase complex formed by ATG13, FIP200, ULK1/2 and ATG101 initiates autophagosome induction. This activated ULK1/2 kinase leads to phosphorylation of beclin-1 complex which consists of beclin-1, class III PI3K, VPS34, VPS15 and ATG14L located at ER to trigger nucleation and generate a double-membrane phagophore. To elongate the phagophore, two ubiquitination-like ATG5 and ATG8 conjugating systems are responsible for LC3(ATG8)-modification to lipidated LC3-II as well as autophagosome expansion. LC3-II is recruited and maintained at autophagosomal membranes and currently serves as a marker of autophagy induction. Several autophagy receptor proteins, such as p62 and NDP52, are able to bind ubiquitinated cargoes as well as LC3-II to sequester cargoes to autophagosomes. Subsequently, autophagosomes fuse with lysosomes by recruitment of specialized SNARE complexes and Rab proteins, resulting in cargo degradation [195, 196].

Accumulated evidence has shown that many intracellular proteins are known to be secreted through autophagy mediated UCPS. For example, autophagic molecular machinery in mammalian cells has been extensively investigated in the study of IL-1β secretion. IL-1β, a proinflammatory cytokine, has been demonstrated to be upregulated in many solid tumors including nasopharyngeal carcinoma, lung cancer, breast cancer and cervical cancer [197]. A significant reduction of IL-1β secretion was observed in *Atg5*−/− macrophages, whereas the induction of autophagy by starvation facilitates its secretion, indicating autophagy-mediated IL-1β secretion in LPS-stimulated macrophages [198]. Furthermore, this autophagy-dependent IL-1β secretion participates in ultraviolet radiation-induced inflammation and skin tumorigenesis [199].

In addition to IL-1β, several intracellular proteins released via secretory autophagy regulate physiological as well as pathological conditions, such as cancer development. Here we discuss the discrepancy between secretory autophagy and degradative autophagy, the molecular mechanisms of secretory autophagy and its contribution in TME.

#### Secretory autophagy vs. degradative autophagy

Autophagy is tightly regulated by *ATG* genes. To date, 42 *ATG* genes have been identified, 15 of which are core *ATG* genes critically regulating autophagic activity. Secretory and degradative autophagy both share the same processes including initiation, nucleation, membrane expansion, autophagosome maturation and amphisome formation. Secretory autophagy leads to the fusion of autophagosomes with late endosomes or MVBs, which in turn form amphisomes for trafficking to PM for the release of cargoes. On the other hand, degradative autophagy promotes amphisomes to fuse with lysosomes to form autolysosomes, and hence degradation of contents occurs for cellular recycling [200].

It is still unclear how cargo-containing vesicles are determined to undergo secretory or degradative autophagy. Although these questions remain elusive, there are some clues indicating the discrepancy between secretory and degradative autophagy. First, proteins usually labelled by ubiquitination are selectively sorted for degradative autophagy through autophagic receptors, such as NDP52, NBR1, OPTN and p62. Autophagic receptors utilize their ubiquitin binding domains to target ubiquitinated proteins and guide them to autophagosomes via their LC3-interacting region (LIR) motifs for the following lysosomal degradation [201]. However, certain autophagy related proteins, including NBR1, p62 and LC3, are found in exosomal fractions of PC3 cells treated with apilimod, an inhibitor of cytosolic 5-phosphoinositide kinase (PIKfyve), for potential antiviral and anti-cancer drug. In addition, polyubiquitination at sites of K11, K48 and K63 is increased in exosomal fractions after apilimod treatment. Inhibition of phosphoinositide kinase PIKfyve enhances both secretion of EVs and autophagy [202]. These findings suggest involvement of the autophagic receptors and ubiquitination in secretory autophagy.

Second, the level of cholesterols in MVBs might play a role in determining cargo-carrying vesicles for degradative or secretory autophagy. By using perfringolysin O to localize cholesterol-rich membranes in human lymphoblastoid cells via electron microscopy, a study revealed that high amounts of cholesterol are found on membranes of those MVBs closed to PM and exosomes, whereas cholesterol-poor MVBs are localized around Golgi cisternae as well as lysosomes [203]. The findings from this study suggest that cholesterol-rich MVBs may favor exosomal transport while cholesterol-poor MVBs may fuse with lysosomes. However, further investigations are required to understand the regulatory mechanisms.

Third, several SNARE proteins and Rab GTPases are revealed to participate in degradative or secretory autophagy. For example, Rab7, STX17 and Rab8b are known to participate in the fusion of autophagosomes with lysosomes for lysosomal degradation in degradative autophagy. On the other hand, Sec22B mediates the fusion of cargo-carrying autophagosomes to MVBs or the PM for cargo secretion via secretory autophagy. In MVB-mediated pathway, MVBs are recruited by Rab11 to fuse with autophagosomes for amphisome formation. Rab8a and Rab27a are responsible for transporting amphisome to the PM. Most cargoes such as Wnt5A, MMP2, IL-6, IL-8, annexin A2, galectin-1, HMGB1, type I collagen and fibronectin, are assumed to be delivered through the MVB-dependent secretory autophagy. In autophagosome-directed secretion, SNAP23/29 and STX3/4 facilitate the fusion of autophagosome with the PM for cargo release (see details in “Interaction with vesicle trafficking systems by secretory autophagy” section).

According to these emerging studies, most of the autophagy-related genes are crucial factors involved in both degradative and secretory autophagy, Furthermore, SNAREs, Rab proteins, cholesterol level, ubiquitination and autophagy receptors are found to participate in regulating these two processes.

#### Interaction with vesicle trafficking systems by secretory autophagy

Endosomal/MVB-mediated exocytosis system has been revealed to participate in secretory autophagy. By fusing with autophagosomes, late endosomes/MVBs can form amphisomes to degrade cargoes via lysosomes or promote cargo secretion by fusing with PM. Releasing of extracellular exosomes occurs when MVBs are fused with PM (see “Subtypes, biogenesis and secretion of EVs” section). This indicates crosstalk between exosome and secretory autophagy. In fact, several lines of evidence point to the regulation of exosome biogenesis by autophagy-related proteins. ATG5 and ATG16L were found to positively increase exosome production by preventing acidification of MVBs. ATG5-ATG16L complex on MVB membrane is able to disrupt V1V0-ATPase via LC3 to reduce MVB lysosomal degradation and hence increases its fusion with PM to release exosomes. This ATG5-mediated exosome production facilitates breast cancer cell migration and metastasis in a mouse model [204]. In addition, another study showed that ATG12–ATG3 complex controls late endosome distribution, EV biogenesis and viral budding via interacting with an ESCRT-associated protein ALIX, a crucial protein in regulating EV biogenesis [205]. These observations illustrate that autophagy is involved in EV biogenesis.

**Fig. 5:**
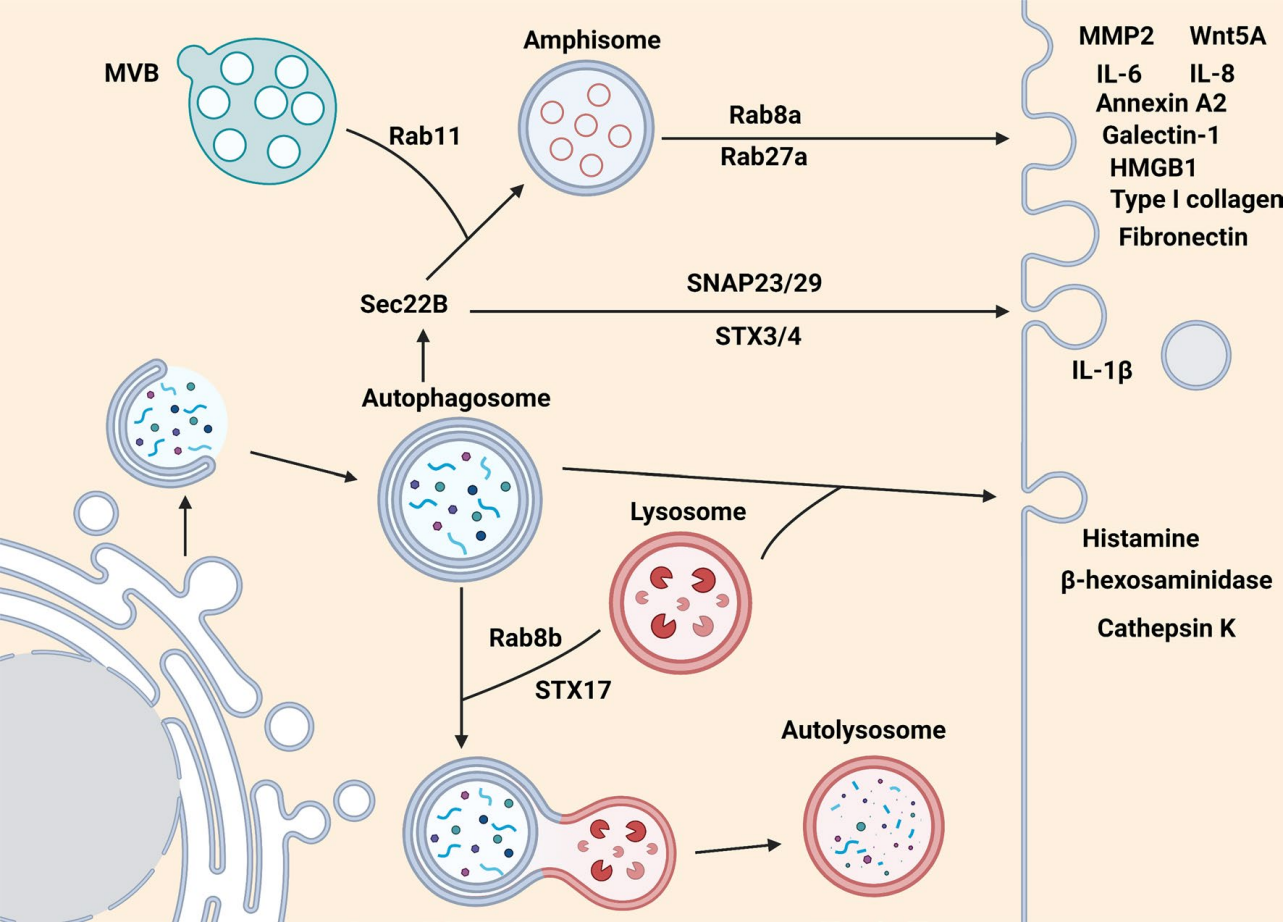
Critical Rab and SNARE proteins participate in degradative and secretory autophagy. In degradative autophagy, Rab8b and STX17 are essential for autophagosomes to attract lysosomes for fusion and form autolysosomes for cargo degradation. On the other hand, three modes of secretory autophagy are proposed to mediate cargo release. First, MVB fuses with autophagosomes to form amphisomes mediated by Rab11 and then transport to PM via Rab8a- and Rab27a-mediated route. Most cargoes such as Wnt5A, MMP2, IL-6, IL-8, annexin A2, galectin-1, HMGB1, type I collagen and fibronectin, are believed to be secreted via MVB-dependent secretory autophagy. Second, IL-1β-carrying autophagosomes can be directly transported to PM with the help of SNAP23/29 and STX3/4. Of note, Sec22B is involved in both the secretory autophagy pathways. The last one is mediated via secretory lysosomes. Autophagosomes fusing with lysosomes are able to facilitate the secretion of histamine, β-hexosaminidase and cathepsin K

Rab GTPase protein family are key regulators responsible for intracellular trafficking of vesicles, including exosomes, MVBs, amphisomes and lysosomes (Fig. 5). For example, activation of Rab7 by starvation is reported to facilitate recruitment of lipid droplets (LDs), LC3+ autophagosomes, MVBs and lysosomes for breakdown of LDs in hepatocytes. This Rab7-mediated LD breakdown requires interaction with its effector Rab-interacting lysosomal protein (RILP) [206]. In addition, Rab8a and Rab8b are two essential factors involved in secretory and degradative autophagy, respectively. By interacting with TBK-1 (TANK-binding kinase 1), Rab8b is responsible for autophagosome maturation that fuses with lysosomes in degradative autophagy [207]. Thus, knockdown of Rab8b impairs the maturation of *Mycobacterium. tuberculosis var. bovis* BCG phagosomes into autophagolysosomes, leading to reduction in bacteria elimination [208]. In contrast to Rab8b, Rab8a is required for autophagy-mediated IL-1β secretion pathway under starvation and inflammasome activation. Loss of Rab8a or overexpression of dominant-negative Rab8a mutant (S22N) inhibits IL-1β secretion via autophagy, indicating that Rab8a plays a crucial role in autophagy-mediated UCPS [209]. Notably, Rab11 is previously reported to be involved in the fusion of MVBs with autophagosomes [210]. Its role in fostering protein release via secretory autophagy has been noticed. For example, Rab11, EEA1 and LC3 on amphisomes play a critical role in promoting mucin secretion via production of reactive oxygen species (ROS) in mouse intestinal goblet cells [211]. A phospholipid-binding protein called annexin A2 (ANXA2) is recently reported to be released to the extracellular matrix via Rab11 in an autophagy-dependent regulation under IFN-γ stimulation in lung cancer cells. In addition, Rab8a and Rab27a are essential for trafficking of ANXA2-carrying amphisomes and their fusion with PM to trigger IFN-γ-induced ANXA2 secretion [212]. These results illustrate that Rab11 participates in the fusion of autophagosomes with MVBs to form amphisomes while Rab8a and Rab27a facilitate the trafficking of these amphisomes to the PM and release of cargoes.

SNAREs are core proteins that mediate fusion of vesicle membranes with target membranes in vesicle trafficking (Fig. 5). It has been shown that SNARE Syntaxin 17 (STX17, soluble *N*-ethylmaleimide-sensitive factor attachment protein receptor) is critical for fusion of autophagosomes and lysosomes in degradative autophagy [213]. STX17 localizes to the outer membrane of autophagosomes for fusion with lysosomes through interacting with SNAP29 and the endosomal/lysosomal SNARE VAMP8. Depletion of STX17 contributes to the accumulation of autophagosomes without lysosomal degradation [214]. On the other hand, R‐SNARE Sec22B, a vesicle trafficking protein, interacts with the secretory autophagy receptor tripartite motif-containing 16 (TRIM16) to facilitate cargo secretion via secretory autophagy. The findings have been observed by investigation on the mechanism of secretion of IL-1β as a cytosolic protein cargo. IL-1β is recognized by TRIM16 together with galectin-8 [215]. The interaction of TRIM16 and Sec22B facilitates the transportation of IL-1β into LC3^+^ sequestration membranes. It has been known that STX17 is essential for maturation of autophagosomes into autolysosomes. Interestingly, Sec22B interacts with STX3 and STX4 instead of STX17 for averting from lysosomal degradation. By interacting with R-SNARE, Sec22B and cooperating with Qbc-SNAREs SNAP23/29 (Qbc, the conserved glutamine (Q) residue at two SNARE motifs) and Qa-SNARE STX3/4 (Qa, a conserved glutamine (Q) residue at one SNARE motif), this allows fusion of IL-1β-carrying autophagosomes with the PM to release IL-1β [215, 216].

Given the biological relevance of shifting between degradation and secretory autophagy during starvation or stress in cell-type-specific context, it is of great interest to identify the specific Rab and SNARE proteins and their effectors or cargoes in these vesicles (Fig. 5). Although several Rab effector proteins, such as RILP and TBK-1, are revealed to participate in degradative autophagy, the effector proteins responsible for secretory autophagy remain unclear. The future studies of the molecular mechanisms underlying how Rab and SNARE specifically regulate secretory autophagy will advance current understanding on how cargoes accumulate in different disease modes or in TME.

### Secretory autophagy in TME

During tumor development, cancer and stromal cells are prone to upregulate autophagic activity to facilitate cancer progression. Beyond the notion of degradative autophagy, secretory autophagy is able to regulate the intercellular communication in TME by mediating protein release. Those proteins secreted from cancer cells or stomal cells can facilitate the establishment of immunosuppressive environment, tumor growth, metastasis and drug resistance in TME.

**Fig. 6:**
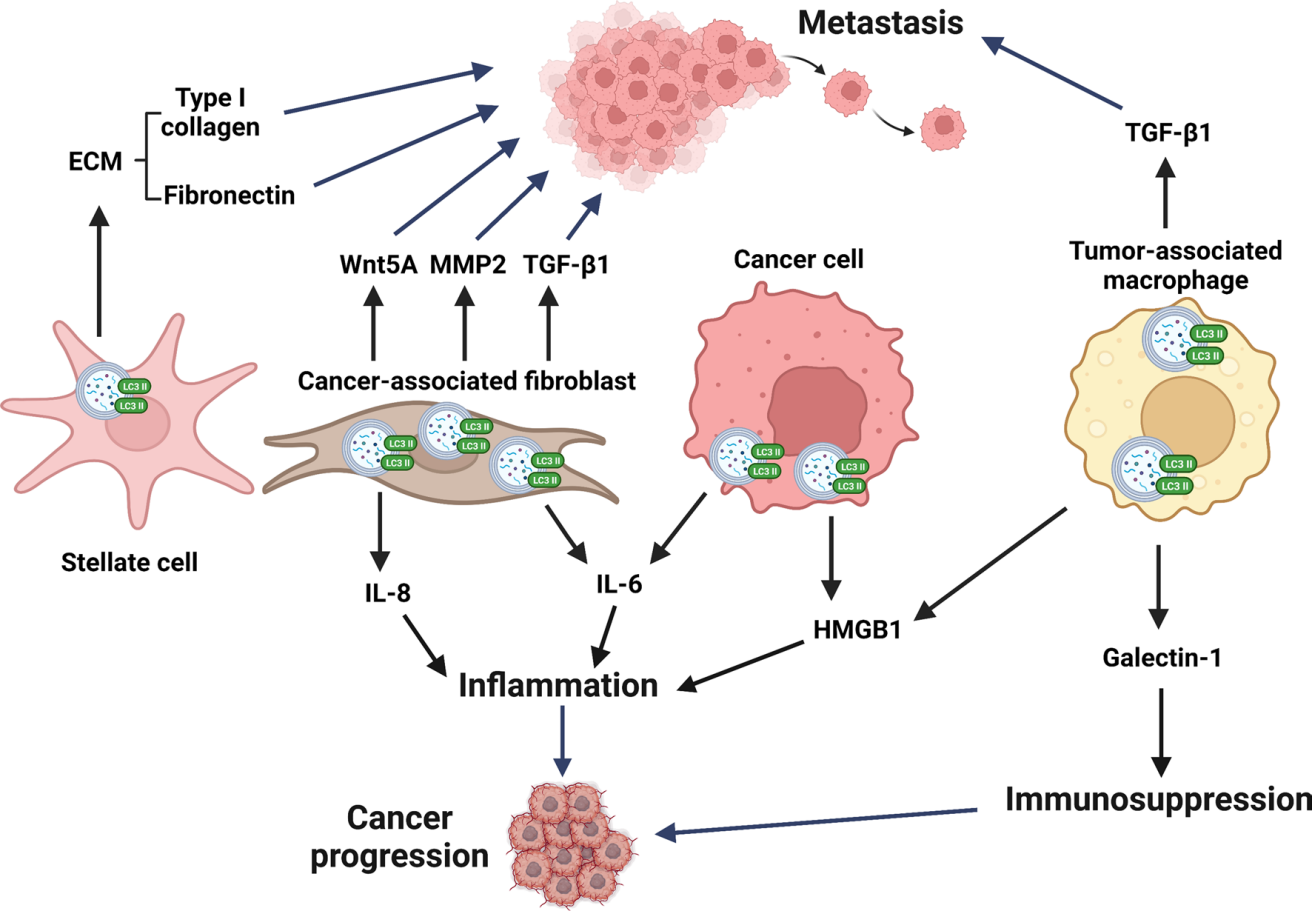
Secretory autophagy mediated release of pro-tumor factors in TME contributes to cancer progression and tumor metastasis. Both cancer and stromal cells are able to release pro-tumor factors via secretory autophagy. Autophagy-mediated release of type I collagen and fibronectin from stellate cells as well as Wnt5A, MMP2 and TGF-β1 from cancer-associated fibroblast (CAF) or TGF-β1 from tumor-associated macrophage (TAM) facilitate tumor metastasis. In addition, autophagy-regulated release of IL-8 and IL-6 derived from CAFs or HMGB1 from TAM and cancer cells leads to inflammation and cancer progression. Furthermore, TAM produced galectin-1 via secretory autophagy creates an immunosuppressive TME and enhances tumor growth, which altogether leads to cancer progression. *ECM* Extracellular matrix

According to the study of autophagy-dependent secretomes in melanoma cells, low autophagic activity contributes to a significant decrease in the serum level of IL-1β, CXCL8, LIF (leukemia inhibitory factor), FAM3C (family with sequence similarity 3, member C) and DKK3 (dickkopf WNT signaling pathway inhibitor 3) compared to melanoma cells with high levels of autophagy [217]. Those secreted proteins play a crucial role in inflammation and tumorigenesis. When melanoma cells with low autophagic activity were treated with autophagy-inducing tat-beclin 1 peptides, secretion levels of those proteins were enhanced. On the contrary, when ATG7 was silenced in cells with high autophagic activity, the levels of secreted proteins were reduced, suggesting that the serum level of autophagy-mediated secretory proteins may be the biomarkers for targeting autophagy in cancers [217]. Here, we discuss the role of secretory autophagy in TME from the aspect of cancer cells and stromal cells (Fig. 6).

#### IL-6

IL-6 is a pro-inflammatory cytokine that participates in chronic inflammatory conditions, which is one of the leading causes of cancer progression. The autophagy-regulated IL-6 secretion in TME has been observed. Inhibition of autophagy by knocking down ATG7 or beclin 1 in breast cancer stem cells diminishes IL-6 secretion, cell survival as well as mammosphere formation [218]. In line with breast cancer stem cells, knockdown of ATG7 in immortalized pancreatic stellate cells reduces not only IL-6 secretion but also the release of ECM proteins such as type I collagen and fibronectin. Autophagy inhibition in immortalized pancreatic stellate cells suppresses tumor growth, liver metastasis and peritoneal dissemination in vivo [219]. Apart from cancer cells, CAFs can also utilize secretory autophagy to manipulate IL-6 secretion. Head and neck squamous cell carcinoma-derived bFGF stimulates the activation of CAFs through FGF receptor signaling and hence facilitates the induction of autophagy. Up-regulation of autophagy in CAFs promotes the secretion of IL-6 and IL-8, whereas autophagy inhibition significantly suppresses release of these cytokines as well as cancer progression [220]. On the other hand, IL-6 can act upstream of autophagy. It has been found that activation of autophagy via IL-6/JAK2/beclin 1 pathway in colorectal cancer [221] in turn facilitates IL-6 secretion by secretory autophagy to promote tumor development and chemotherapy resistance (*Center*, Fig. 6).

#### HMGB1

High mobility group box 1 (HMGB1) is one of proinflammatory alarmins or DAMP (damage-associated molecular pattern) molecules normally localized in the nucleus and acts as a danger signal to warn the surrounding cells when it is secreted extracellularly. In established tumors, HMGB1 released by tumor cells can exacerbate inflammation to promote tumor growth. For example, extracellular HMGB1 stimulates the release of pro-inflammatory cytokines such as IL-6 and IL-8 by triggering MAPK- and MyD88-dependent NF-κB pathways, subsequently facilitating cancer cell proliferation, angiogenesis, EMT, invasion and metastasis [222]. Passive release of HMGB1 usually occurs in cells that are undergoing necrotic cell death [222]. However, active secretion of HMGB1 has been uncovered in tumor cells. It has been found that knockdown of ATG5, ATG7 or ATG12 blocked HMGB1 secretion in diphtheria toxin-treated glioblastoma cells without causing membrane lysis and necrosis, indicating that secretory autophagy is responsible for HMGB1 secretion [223]. In addition to cancer cells, bone marrow-derived macrophages also promote HMGB1 release via secretory autophagy. Upon stimulation of macrophages with nigericin, the delivery of HMGB1 to cytosol was substantially diminished in *Atg5*−/− bone marrow-derived macrophages [198] (*Right*, Fig. 6).

#### Galectin-1

Galectin-1 belongs to a family of β-galactoside-binding lectins and is widely expressed in various tissues. By binding to its glycosylated ligands, extracellular galectin-1 is able to regulate tissue development, immune system activation, pathogen infection and cancer progression. In fact, overexpression of galectin-1 in cancer cells and/or stromal cells is observed to be correlated with poor prognosis in many types of cancers [224]. Since galectin-1 contains no recognizable signal sequence to export via ER-Golgi pathway, the secretion mode of galecin-1 is suggested to be via UCPS. It is found that galectin-1 can be secreted by tumor-associated macrophages (TAMs) via secretory autophagy, which is strongly associated with hepatocellular carcinoma progression. Mechanistically, TLR2-mediated ROS signaling is required for both autophagy induction and galectin-1 secretion via autophagy-based secretion. Galectin-1-carrying autophagosomes fuse with MVBs via Rab11 and VAMP7-mediated vesicle trafficking prior to secretion. This autophagy-regulated galecin-1 secretion promotes tumor growth in mice and correlates to poor prognosis of hepatocellular carcinoma patients. The findings demonstrate the contribution of galectin-1 released by secretory autophagy in TAMs and its role in liver cancer progression [225] (*Right*, Fig. 6).

In addition to galectin-1, secretion of galectin-3 is also regulated by autophagy. Similar to galectin-1, no signal peptide is found in galectin-3 to pass through ER-Golgi route for its secretion. By analysis of secretome data from β-glucan-stimulated macrophages, a recent study revealed that activation of dentin-1 pathway stimulates galectin-3 secretion via inflammasome activity and an active autophagic process. Silencing beclin-1 or 3-methyladenine (3-MA) resulted in decreased release of galectin-3 in dectin-1-activated macrophages, indicating that secretory autophagy may participate in galectin-3 secretion [226]. Elevated expression of galectin-3 in cancer progression has been observed and found to contribute to cancer growth, invasion, migration, angiogenesis and immunosuppression of TME. Of note, galectin-3 can bind T cell receptor to limit T cell movement and signaling pathway, leading to down-regulation of anti-tumor immune response. Targeting galectin-3 becomes a potential therapeutic to improve immune responses against cancer [227]. Based on these current observations, it is worthy to explore the contribution of autophagy-mediated galectin-3 secretion in TME.

#### TGF-β1

Transforming growth factor-β (TGF-β) is a cytokine that regulates numerous cellular functions including proliferation, apoptosis, differentiation, EMT and migration and has been implicated in many diseases such as vascular diseases and cancers. Binding of TGF-β with its receptor induces phosphorylation of serine/threonine residues and triggers phosphorylation of the intracellular effectors, SMADs. Upon activation, SMAD proteins translocate into the nucleus and induce transcription of their target genes, regulating several cellular functions. Dysregulation of TGF-β1 acts as a tumor promoter that stimulates cell migration and EMT in the late stage of tumor development. Elevated expression of TGF-β is associated with poor clinical outcome in some cancers [228]. In addition to cancer cells, TGF-β is abundantly produced by fibroblasts which are critical pro-tumor stromal cells in TME. The secretion of TGF-β in these fibroblasts has been recently found to be regulated by autophagy. This is triggered by an integrin-linked kinase (ILK) dependent signal in fibroblasts. Upon activation of ILK, TGF-β1 was found to interact with GORASP2/GRASP55 in autophagosomes for secretion via Rab8a-dependent pathway. Silencing ATG5, ATG7 and beclin-1 or the treatment with PtdIns3K inhibitor 3-MA significantly abrogated TGF-β1 release from fibroblasts. This autophagy-regulated TGF-β1 secretion is also observed in macrophages. Autophagy inhibition by silencing ATG7 or 3-MA treatment in macrophages impaired TGF-β1 release [229]. These findings indicate secretory autophagy plays a role in facilitating extracellular TGF-β1 release from stomal cells in TME, such as fibroblasts or macrophages, leading to cancer progression and metastasis (*Upper right*, Fig. 6).

#### Matrix metalloproteinase 2 and Wnt5A

Wnt5A, a secreted protein, is regarded as a ligand that triggers non-canonical Wnt pathway in regulating cell proliferation, differentiation, planar cell polarity, adhesion and motility. Non-canonical Wnt signaling transduce a β-catenin-independent transcriptional activity and has been highlighted for its pro-tumor function in tumor development. For example, bone marrow stromal cells can produce Wnt5A to attract prostate cancer cells to migrate into the bone [230]. This indicates that Wnt5A acts as a chemoattractant to promote metastasis of prostate cancer cells. On the other hand, MMP2 is highly expressed and released in various types of cancers, such as colorectal cancer, lung cancer and prostate cancer, and is positively correlated with their progression [231–233]. The secretion modes of pro-tumor factors Wnt5A and MMP2 are controlled by autophagy in RAS transformed cells. It has been demonstrated that knockdown of ATG7 or ATG12 showed a reduction of MMP2 and Wnt5A in the conditioned media in oncogenic RAS transformed cells. Furthermore, this autophagy regulated MMP2 and Wnt5A secretion is essential for cell invasion, migration and lung metastasis in a mouse model [234] (*Upper left*, Fig. 6). These results suggest that autophagy is responsible for the production of multiple secreted factors that are able to increase cancer cell invasion in RAS transformed cells.

In conclusion, these findings suggest that autophagy-based secretion of various tumor-promoting factors from cancer and/or stromal cells modulates TME to promote tumor growth, angiogenesis, metastasis and chemoresistance.

## Conclusions and future perspective

Conventional and unconventional vesicular secretion pathways in TME is crucial for tumor progression. Dysregulation of these conventional vesicle trafficking CPS and unconventional vesicular secretion UCPS pathways is associated with a spectrum of human afflictions, including cancer. CPS and UCPS have been intensively studied since their discovery and recent advances reveal their crucial roles in remodeling TME. These studies showed that the complexity and pleiotropism of CPS and UCPS pathways largely reflect the broad repertoire of cargo substrates and effector proteins. Only a small proportion of these cargoes and effectors have been thoroughly characterized in TME, which suggests that more surprises are yet to come. In this regard, we note two areas where our limited knowledge warrants a closer look. First, we lack a systems-level understanding of how the distinct CPS- and UCPS-mediated cell responses are coordinated in TME. Additionally, it will be important to address how other signaling pathways systemically interact with conventional vesicle trafficking (CPS) and unconventional vesicular secretion pathways (UCPS) including EV and secretory autophagy. Second, future investigation into the spatial and temporal regulation of CPS and UCPS pathways in TME is urgently needed. The development of optogenetic switches and biosensors capable of manipulating and reporting on vesicular trafficking in real time with spatial resolution is currently underway in many laboratories. Coupled with other time-resolved single-cell quantitative measurements of subcellular localizations, we foresee that the coming years will lead to a renewed comprehension of how CPS and UCPS including EV and secretory autophagy shape cell communications within TME. At present, there is no clinical trial specifically on Rab GTPase regulation and secretory autophagy in TME modulation and cancer therapy. Most of the ongoing trials are evaluating diagnostic and therapeutic applications of EVs (www.clinicaltrials.gov accessed on 14 July 2022). Advanced knowledge with spatial and temporal regulation of CPS and UCPS pathways and their cargoes and effectors in TME will strongly influence our ability to develop new cancer therapeutics.

## Data Availability

Not applicable.

## Abbreviations

3-MA: 3-Methyladenine
AICD: Activation-induced cell death
ALIX: ALG-2 interacting protein X
ANXA2: Annexin A2
AP2: Adaptin-2
APC: Antigen-presenting cell
CAF: Cancer-associated fibroblast
CHI3L1: Chitinase 3-like-1
CPS: Conventional protein secretion
CTL: Cytotoxic T lymphocyte
DAMP: Damage-associated molecular pattern
DC: Dendritic cell
DKK3: Dickkopf WNT signaling pathway inhibitor 3
EGF: Epidermal growth factor
EGFR: Epidermal growth factor receptor
EMT: Epithelial–mesenchymal transition
ER: Endoplasmic reticulum
ESCRT: Endosomal sorting complex required for transport
EV: Extracellular vesicle
FAK: Focal adhesion kinase
FAM3C: Family with sequence similarity 3, member C
FGF: Fibroblast growth factor
GAP: GTPase activating protein
GEF: Guanine exchange factor
Golgi: Golgi apparatus
HIF: Hypoxia-inducible factor
HMGB1: High mobility group box 1
HSP: Heat shock protein
IFN: Interferon
IGF: Insulin-like growth factor
ILV: Intraluminal vesicle
IRF3: Interferon regulatory transcription factor 3
LAT: Linker for activated T cells
LIF: Leukemia inhibitory factor
LIR: LC3-interacting region
MDCK: Madin–Darby canine kidney
MDSC: Myeloid-derived suppressor cell
MHC: Major histocompatibility complex
MMP: Matrix metalloproteinase
MSC: Mesenchymal stem cell
MVB: Multivesicular body
PAMP: Pathogen associated molecular pattern
PDGFR: Platelet-derived growth factor receptor
PM: Plasma membrane
PRR: Pattern recognition receptor
PtdIns4,5P2: Phosphatidylinositol 4,5-bisphosphate
Q: Glutamine
RCP: Rab coupling protein
Rin2: Rab interactor 2
SFRP1: Secreted frizzled-related protein-1
SHH: Sonic hedgehog
Slp1: Synaptotagmin-like protein1
STN: Syntenin
STX: Syntaxin
TAM: Tumor-associated macrophage
TBK-1: TANK-binding kinase 1
TCR: T cell receptor
TfR: Transferrin receptor
TGN: Trans-Golgi network
TIIC: Tumor-infiltrating immune cell
TIMP1: Tissue inhibitor of metalloproteinase 1
TLR: Toll-like receptor
TME: Tumor microenvironment
TRAM: TRIF-related adaptor molecular
Treg: Regulatory T cell
TRIF: TIR-domain-containing adapter-inducing interferon-β
TRIM16: Tripartite motif-containing 16
TSG101: Tumor susceptibility 101
TSP1: Thrombospondin 1
UCPS: Unconventional pathways of protein secretion
USP28: Ubiquitin-specific peptidase 28
VEGF: Vascular endothelial growth factor
VPS34: Vacuolar protein sorting 34
